# A secreted proteomic footprint for stem cell pluripotency

**DOI:** 10.1371/journal.pone.0299365

**Published:** 2024-06-14

**Authors:** Philip A. Lewis, Edina Silajdžić, Helen Smith, Nicola Bates, Christopher A. Smith, Fabrizio E. Mancini, David Knight, Chris Denning, Daniel R. Brison, Susan J. Kimber

**Affiliations:** 1 Division of Cell Matrix Biology and Regenerative Medicine, Faculty of Biology, Medicine and Health, University of Manchester, Manchester Academic Health Science Centre, Manchester, United Kingdom; 2 Biodiscovery Institute, Division of Cancer & Stem Cells, School of Medicine, University of Nottingham, University Park, Nottingham, United Kingdom; 3 Royal Manchester Children’s Hospital, Manchester, United Kingdom; Okayama University: Okayama Daigaku, JAPAN

## Abstract

With a view to developing a much-needed non-invasive method for monitoring the healthy pluripotent state of human stem cells in culture, we undertook proteomic analysis of the waste medium from cultured embryonic (Man-13) and induced (Rebl.PAT) human pluripotent stem cells (hPSCs). Cells were grown in E8 medium to maintain pluripotency, and then transferred to FGF2 and TGFβ deficient E6 media for 48 hours to replicate an early, undirected dissolution of pluripotency. We identified a distinct proteomic footprint associated with early loss of pluripotency in both hPSC lines, and a strong correlation with changes in the transcriptome. We demonstrate that multiplexing of four E8- against four E6- enriched secretome biomarkers provides a robust, diagnostic metric for the pluripotent state. These biomarkers were further confirmed by Western blotting which demonstrated consistent correlation with the pluripotent state across cell lines, and in response to a recovery assay.

## Introduction

Although the clinical potential of regenerative medicine, including approaches using human pluripotent stem cells (hPSCs), remains unrivalled, major hurdles exist in translating promising protocols for treatments into scalable, reproducible commercial processes. Maintaining hPSCs in their pluripotent state has proved challenging and labour intensive. Advances have been made in automating the culture and expansion of high quality hPSCs, such as by the development of the SelecT (TAP biosystems—Hertfordshire, UK), Freedom EVO (TECAN Trading AG, Switzerland) systems, or technologies developed by Tokyo Electron (Kyoto, Japan). However, a key issue in hPSC culture that remains to be overcome is the absence of a rapid, reproducible, non-invasive, and quantitative metric for pluripotent stem cells.

All of the most commonly used techniques have major drawbacks when it comes to their use in hPSC production. For instance, using immunofluorescence for assessing the core pluripotency transcription factors and cell surface markers does not accurately pick-up initial loss of pluripotency in entire cultures, but rather relatively late loss of pluripotency in individual cells. Immunofluorescence is also imprecise, generally non-quantitative, cannot easily be integrated into the manufacturing process, and the tested cells are lost from culture in the assay. Using flow cytometry for identifying loss of pluripotency markers is helpful but shows profound run variability and again sacrifices cell product. |At present the most quantifiable metrics of pluripotency available are RNA-based assays on cells, such as. Pluri-test [1] and ScoreCard [2], or teratoma assays [3]. However, these metrics are costly and require sacrifice of pluripotent cell product. Moreover, they also take too long to provide results for ‘at-risk’ cultures, which would likely become irreversibly compromised by the time the results are available. The most rapid and cost-effective tool researchers currently have is analysis of cell morphology, however this is inherently subjective. While attempts have been made to automate and quantify the characteristics of hPSC morphology [4, 5], it remains a low-resolution way to measure pluripotency [6]. Thus, there are no fit for purpose quantitative assays which can rapidly pick up very early loss of pluripotency.

To address these limitations, we sought to identify secreted protein biomarkers in conditioned culture medium within 48 hours of the onset of hPSC pluripotency dissolution. Conditioned medium would be particularly suitable for inline monitoring in a commercial setting such as for therapeutic manufacture, allowing regular, rapid, repeated media sampling for MS or other evaluation without disturbing the growth of the culture. Although the secretome of hPSCs has been studied in the past [7–11], there is yet to be a comparative study aiming to identify proteins in medium indicative of the pluripotent state, and its loss. It is well established that hPSC self-renewal relies on the signalling pathways down stream of FGF-2 and TGFβ family members [12–14]. After removal of these growth factors from standard hPSC culture medium the cells lose their core pluripotency network, a prerequisite for lineage differentiation [15]. Therefore, we used the removal of FGF-2 and TGFβ from the medium to trigger loss of the pluripotent state, aiming to identify protein biomarkers which could be detected in conditioned medium while it is still possible to rescue the stem cell state. Using LC-MS/MS (Liquid Chromatography with tandem Mass Spectrometry) of conditioned medium from two pluripotent stem cell lines (one hESC, one hiPSC), we identified 117 secreted proteins which were highly significantly changed in their abundance (q<0.01) within 48 hours of E8 medium being substituted for the FGF-2/TGFβ deficient E6. Of these 74 were identified as lower in E6 medium than in E8, and 38 higher.

Additionally, we address the fundamental limitation in the use of secreted proteins in media as biomarkers, that the concentration of such proteins will vary greatly dependent upon medium volume, cell concentration, timing of passage and cell health, in a way which cannot routinely be normalised for. As such we identified a panel of biomarkers which were highly significantly changed in both cell lines (q<0.05) in secreted medium and, for all but one marker, also at the RNA level. The relative abundances of these biomarkers when multiplexed provide a more robust metric for this change than the individual protein abundances. We propose that the use of protein abundance ratios provides a more reliable metric for pluripotency loss that is not affected by changes in overall protein concentration in the media caused by cell seeding and variability in cell viability and health, or applied medium volume. We further demonstrate the utility of these markers by returning E6 cultures to E8 medium, leading to a full recovery of pluripotency. Such indicators in the conditioned medium are amenable to simple detection assays and will be particularly useful in a scale up culture setting to prevent or reverse culture deterioration while avoiding loss of product.

## Results

### Incipient pluripotency loss after 48 hours of FGF2/TGFβ removal is at the limits of detection by flow cytometry and immunocytochemistry

**Fig 1:**
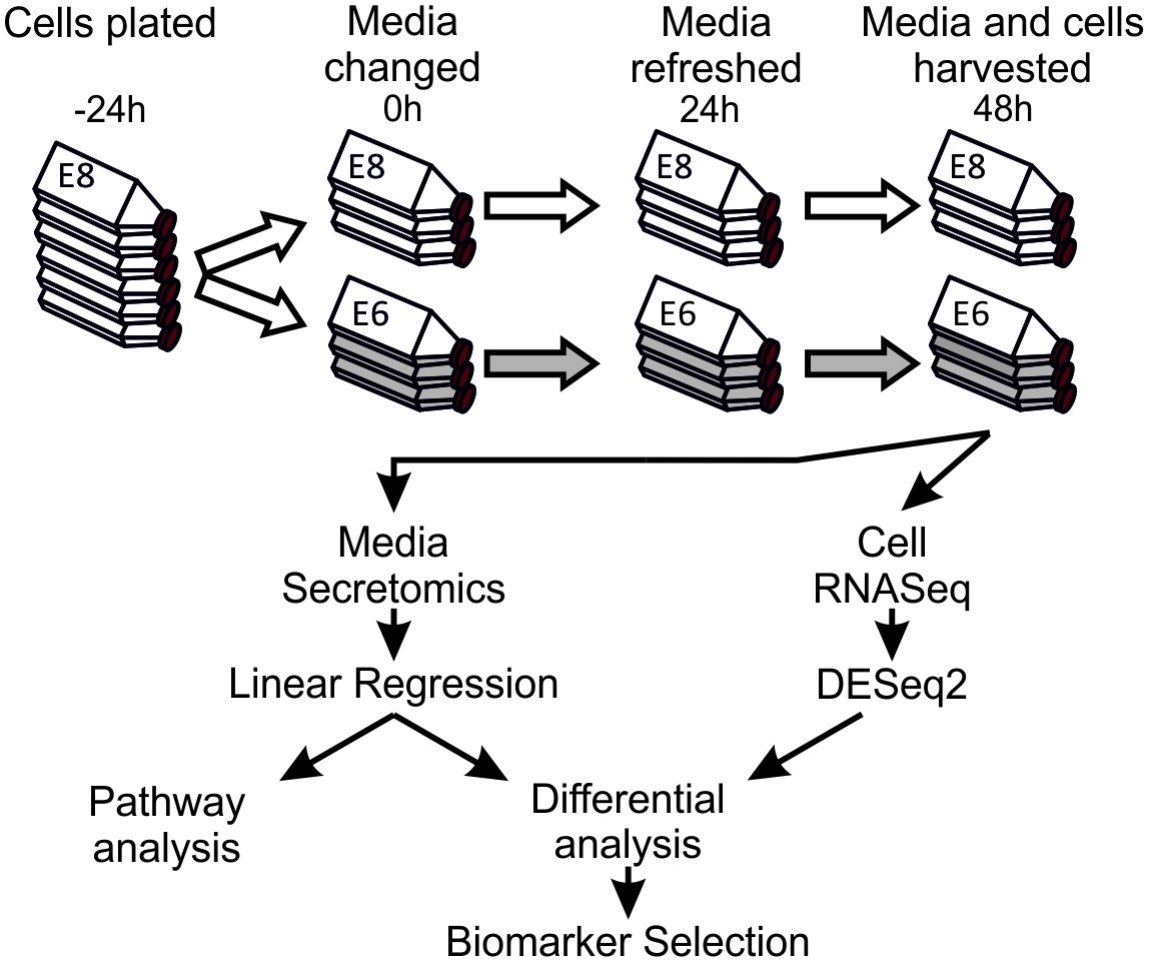
Experimental design for sample collection and data processing. Cells were cultured in E8 for at least two passages prior to the onset of the experiment. Cells were then plated in E8 medium containing Rock inhibitor onto Vitronectin-N coated flasks and were allowed to settle for 24 hours before onset of experimental conditions. Cells were washed in PBS, then half were cultured in E8, and half in E6, with full medium replacement after 24 hours. After 48 hours of culture under experimental conditions the medium was collected, spun, concentrated, and processed for LC-MS/MS. Cells were also collected and processed for quality control, and RNA-Seq analysis.

We first confirmed the time scale of loss of conventional human pluripotency-associated markers. HESC (Man-13: [16]) and hiPSC (Rebl.PAT: [17]) lines (collectively referred to as hPSCs) were cultured in either pluripotency maintenance medium (E8 [18]), or identical medium lacking the pluripotency maintenance factors FGF2 and TGFβ1 (E6 [19]) for 48 hours, before analysis (Fig 1). A previous study indicated that mRNA for lineage specific marker proteins is not definitively expressed until at least four days in E6. However, loss of pluripotency-associated markers was detectible after as little as 48 hours following removal of FGF2/TGFβ [15]. Therefore, it was hypothesised that it should be possible to identify secretome changes associated with the loss of pluripotency at this early time point, enabling cultures to be identified at a point at which they can be rescued. In this manuscript we use the word secretome to refer to all proteins identified in the conditioned media in both cell lines, which may include proteins shed through means beyond canonical cell secretion pathways.

In E8 or 48 hours after FGF2/TGFβ removal (in E6), MAN13 cells exhibited broadly comparable immunofluorescence and staining for pluripotency markers OCT4 and SSEA3. In Man-13 cells cultured in E6 for 48 hours there was a slight decrease in immunofluorescence for NANOG and TRA160, and appearance of the early differentiation marker, SSEA1 (S1A and S1B Fig). Flow cytometric data shows similar NANOG distribution in both Man-13 and Rebl.PAT cells grown for 48 hours in E6 compared to E8 medium, whereas TRA1-81 marginally decreased in Rebl.PAT cells and marginally increased in Man-13 cells (S1B Fig). Only SOX2 was consistently observed to be moderately increased after 48 hours in E6 (S1A and S1B Fig) confirming transcript data (S1C Fig). At 48hours, *OCT4* was observed to be slightly increased, as is common on initial differentiation, at the transcript level in Rebl.PAT and by flow cytometry in Man-13 (S1B and S1C Fig). Additionally, no significant change in viability of cells between E8 and E6 was observed (S2 Fig).

Although it is well established that feeder-free pluripotent stem cell maintenance requires FGF2 and TGFβ, without which cells lose pluripotency, our results indicate that current standard methods cannot unambiguously detect the incipient loss of pluripotency 48h after growth factor withdrawal. Whilst routine flow cytometry, RT-PCR and immunofluorescence can identify some subtle changes in pluripotency-associated markers at this time, the changes are only distinguishable when comparing these samples with high quality, stable pluripotent controls cultured in parallel. In isolation these changes would be unreliable if used to identify incipient pluripotency loss.

### Consistent, detectable changes in the secretome are observable after 48 hours of FGF2/TGFβ removal

**Fig 2:**
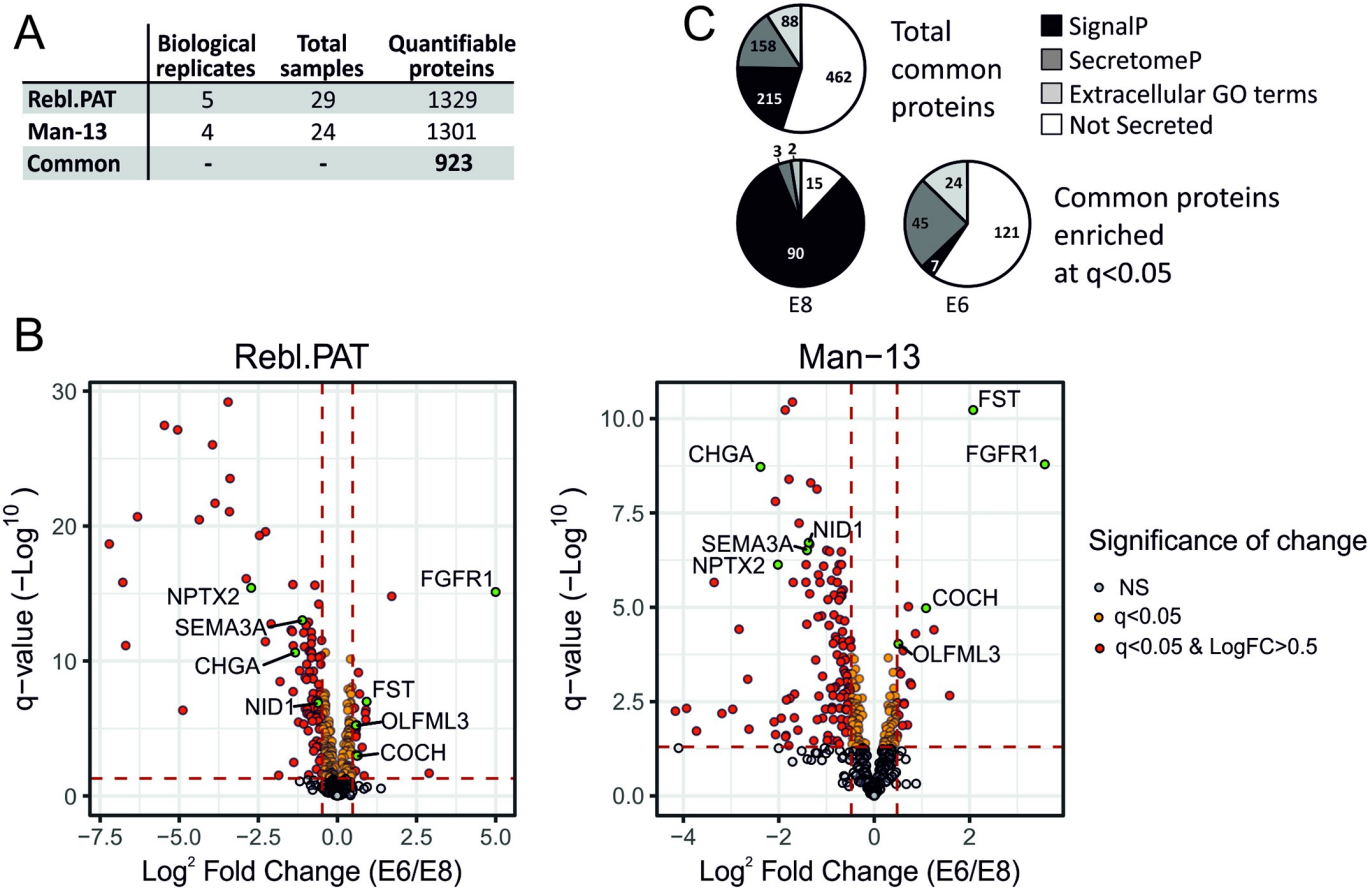
Proteomics summary. A) Summary of the proteomics experimental inputs and outputs. B) Pie charts demonstrating the proportion of proteins which are identified as secreted by (in order of priority) SignalP 5.0, SecretomeP 2.0 or by extracellular Gene Ontology (GO) terms associated with their Uniprot accessions. Extracellular GO terms used for classification of proteins were extracellular matrix, extracellular space, extracellular vesicle, cell surface and extracellular region. All proteins used for this analysis had one or more unique peptides, and a Progenesis confidence score of 30 or more in all three proteomics runs. C) Volcano plots of secretomic datasets. Proteins q>0.05 are shown in grey, q<0.05 shown in yellow and q<0.05 + LogFC>0.5 shown in red. Proteins shown in green are the proteins identified as secreted pluripotency marker proteins.

Medium conditioned by stem cells cultured in either E8 or E6 was collected according to the workflow shown in Fig 1, after 24 and 48 hours. Briefly, equal volumes of media were spun to remove any floating cells and debris, then concentrated in 10kDa centrifugal filter units, before 50ug (Man-13) or 25ug (Rebl.PAT) of protein from each sample was prepared for label-free LC-MS/MS using by Filter-Assisted Sample Preparation (FASP). Acquired MS data for each MS experiment were analysed in Progenesis LC-MS and peak lists were searched against Swissprot/Trembl database using Mascot v2.5.1 to give protein abundance measurements. In total we analysed 29 Rebl.PAT samples (5 batches of 3 biological replicate flasks for E8 & E6, 1 QC failure removed) and 24 Man-13 samples (4 batches of 3 biological replicate flasks for E8 & E6, 1 QC failure removed) by label-free LC-MS/MS, identifying a total of 923 common proteins detected at quantifiable levels (a Progenesis confidence score of >30) in both cell lines (Fig 2A). Principal Component Analysis (PCA) of the proteomics data correctly separated the samples by culture medium type. However, it also identified the cell lines, and the MS batch effects as strong sources of variance (S3 Fig). As such differential analysis of the protein abundance between E8 and E6 samples was performed separately for each cell line using linear regression models, fitted independently for each protein using protein abundance as the dependent variable, and medium as the independent variable, with biological replicate included as a covariate. A common trend in both cell lines was that although protein abundance was normalised to total protein amount, a larger number of proteins were significantly enriched (q<0.05) in E8 than in E6 (Fig 2B). We also identified that 84% of E8-enriched proteins (q<0.05) were secreted, as identified by the presence of an amino-acid secretion tag (identification through SignalP [20]), or by an amino-acid sequence indicative of non-classical secretion (identification through SecretomeP [21]). By contrast, a method of secretion could not be identified for 64% of E6 enriched proteins, including by presence of annotation using the cellular component gene ontology (GO) tags; extracellular space, extracellular vesicle, cell surface, or extracellular region (Fig 2C).

### Correlation between changes in protein and RNA abundance

**Fig 3:**
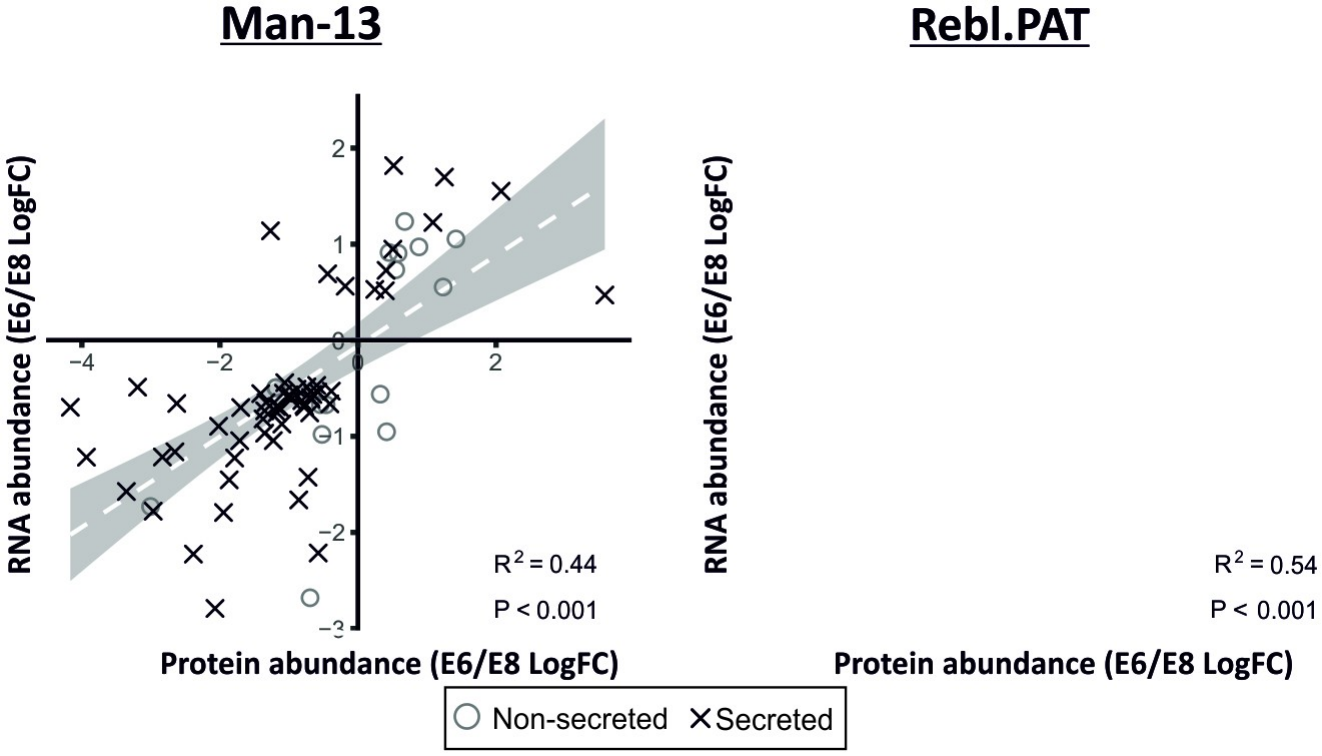
Correlation between E6 /E8 log fold-changes between mRNA and protein data. Protein data was paired to RNA-Seq data using Entrez IDs retrieved from Uniprot and Bioconductor [22–24]. These data show a correlation between the changes observed in significantly changed (q<0.05) secreted proteins and the corresponding RNA-seq data (Man-13 R^2^ = 0.44 & Rebl.PAT R^2^ = 0.54).

RNASeq was performed on cells from a selection of the replicates to investigate whether changes in secretome correlated with changes at the transcript level (Fig 3). We observed a significant positive correlation between the LogFCs of significantly changed (q<0.05) secreted proteins and RNA transcripts (M13 R^2^ = 0.44, p<0.001; Rebl.PAT R^2^ = 0.54, p<0.001). Further investigation into the minority of proteins which correlated negatively identified that these tended not to be identified as secreted.

### Pathway analysis

**Fig 4:**
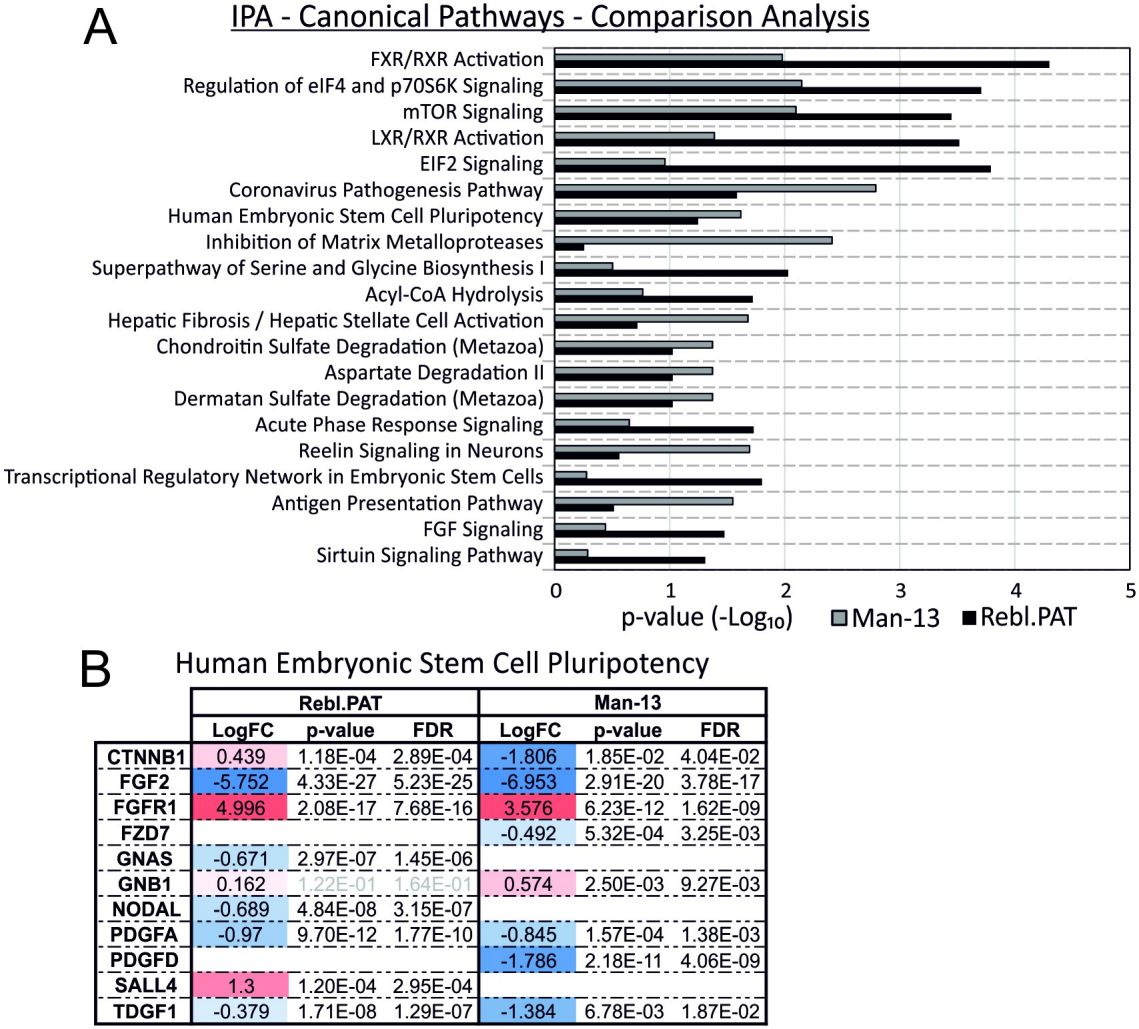
Canonical Pathways identified by Ingenuity Pathway Analysis (IPA) of secreted proteomics data. A) Independent pathway analyses were performed for Man-13 and Rebl.PAT comparing proteins with a q<0.05 change between E6/E8 to total secretome of each cell line. A comparison analysis is shown of the top canonical pathways identified as significantly enriched in both cell lines. B) Individual proteins identified as changed in the Human Embryonic Stem Cell Pluripotency pathway for both Man-13 and Rebl.PAT.

Secretomic data were uploaded to Ingenuity Pathway Analysis (IPA) for pathway analysis of significantly changed proteins (q<0.01, >1.4FC). Though in-depth pathway analysis on secreted proteomics data is limited in its utility as large portions of most signalling pathways are intracellular, and as such not detectable in this assay, IPA analysis identified elements of the Human Embryonic Stem Cell Pluripotency pathway were significantly enriched in both cell lines (Fig 4).

### Selection of biomarker panel

As human PSC culture media contain high concentrations of serum proteins such as Transferrin, or Serum Albumin, the proportion of proteins in conditioned medium that originates from cells can vary dramatically dependent upon variances in culture volume, evaporation, seeding density, starting viability, or growth rate. For the presented assays we aimed to limit the effect of these variables, however we acknowledge that they present a limitation to the accurate relative quantification of secreted protein abundance by affecting normalisation to total protein abundance. To overcome this, we sought to identify a biomarker panel which would allow for discrimination between sample types in a robust fashion and which could be quantified without having to normalise to total protein quantity.

As biomarkers which both increase and decrease during loss of pluripotency are responding to the same stimulus but in different directions, we anticipate that the change in their relative abundance upon loss of pluripotency should be consistent, regardless of variance in total protein concentration. Therefore, we sought to identify a marker set which could be internally normalised against each other rather than relying on volume or total protein abundance normalisation.

**Fig 5:**
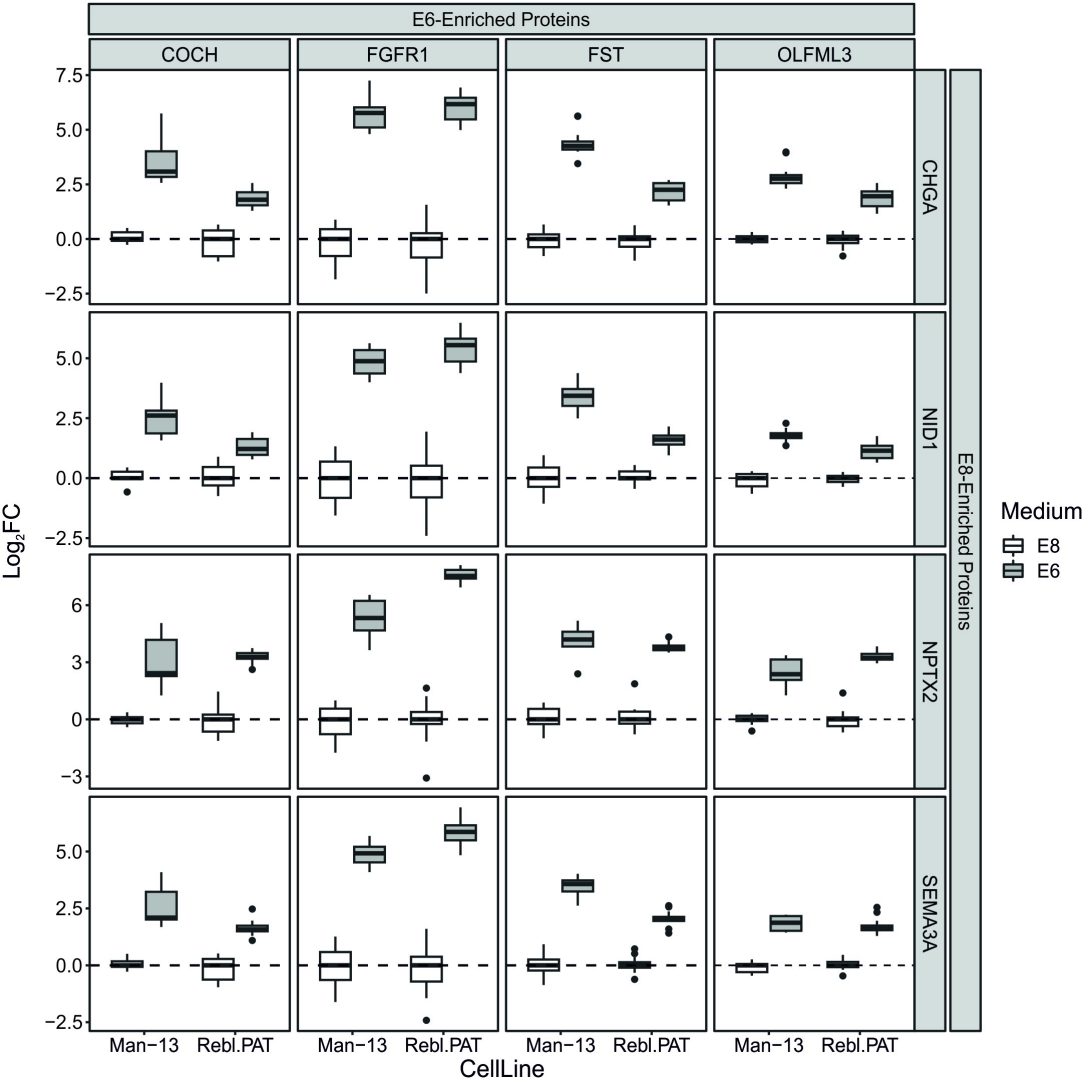
Box plots demonstrating efficacy of multiplexing marker proteins. For each boxplot, the LogFCs of two protein abundances were calculated independently for each sample. These LogFCs are calculated from two marker proteins, one identified as enriched in E6 (top) over one identified as enriched in E8 (right). By normalising the protein abundances against each other in this way we are able to demonstrate the consistent changes in the relative abundance of these proteins in all combinations of markers, and all samples across both cell lines. For ease of comparison across different protein combinations, these LogFCs are scaled such that the median LogFC in samples grown in E8 media is 0. For every marker protein combination, the difference between mean ratios for E8 and E6 media was below p<1E-8 & >1 LogFC.

We selected a list of secreted proteins which demonstrated highly consistent changes in both RNA and protein abundance between conditions, and tested whether the relative abundances of these proteins formed ratios which were consistent and discriminatory between E8 and E6 conditions, thus, they should be robust irrespective of the normalisation challenges of conditioned medium (Table 1). From this list, the relative abundance of each E8-enriched protein compared to each E6-enriched protein was calculated and the resultant ratios were filtered for those which demonstrated the most statistical separation between culture media (p<1E-8 & >1 LogFC). This resulted in a panel of four proteins which increased in medium when cells were cultured in E6 compared with E8—Cochlin (COCH), FGF Receptor-1 (FGFR1), Follistatin (FST) and Olfactomedin-like 3 (OLFML3)-, and four proteins which were higher when cells were cultured in E8 compared E6 –Chromogranin A (CHGA), Nidogen 1 (NID1), Neuronal Pentraxin-2 (NPTX2) and Semaphorin 3A (SEMA3A). The relative abundances of these proteins provide an internally normalised set of ratios which each discriminate between E8 and E6 medium after 48 hours, in a manner which avoids the variables which thwart quantification of conditioned medium biomarkers (Fig 5).

**Table 1:** Secreted proteins significantly changed in both RNA and protein abundances in both Rebl.PAT and Man-13 cell lines.

		LogFC (E6/E8)				Adj. P-value			
		Protein		RNA		Protein		RNA	
		Man-13	Rebl. PAT	Man-13	Rebl. PAT	Man-13	Rebl.PAT	Man-13	Rebl.PAT
**E6-Enriched**	**COCH**	1.09	0.62	1.23	0.54	1.05E-05	1.06E-03	1.02E-10	5.17E-06
	**FGFR1**	3.58	5.00	0.47	0.24	1.62E-09	7.68E-16	2.57E-02	8.59E-02
	**FST**	2.07	0.92	1.55	1.04	5.91E-11	1.03E-07	1.50E-18	9.79E-24
	**OLFML3**	0.51	0.58	0.95	0.76	9.31E-05	5.93E-06	4.25E-07	1.47E-03
	**SERPINB9**	1.25	1.71	1.70	1.89	3.95E-05	1.61E-15	1.91E-07	1.90E-22
**E8-Enriched**	**ADAMTS8**	-3.36	-0.42	-1.57	-0.66	2.19E-06	2.23E-03	1.65E-20	2.14E-06
	**APP**	-1.00	-0.71	-0.57	-0.43	3.09E-07	2.43E-16	2.73E-03	7.73E-06
	**B3GNT7**	-1.17	-0.81	-0.75	-0.62	1.38E-06	2.09E-12	1.20E-03	7.72E-09
	**CHGA**	-2.38	-1.34	-2.23	-1.37	1.89E-09	2.34E-11	7.75E-31	2.50E-21
	**COL5A2**	-2.65	-0.89	-1.16	-0.80	8.07E-04	8.52E-07	1.95E-09	8.06E-16
	**EFEMP1**	-2.83	-1.07	-1.22	-0.67	3.83E-05	8.08E-13	9.72E-10	3.72E-12
	**EPHA1**	-1.33	-0.61	-0.65	-0.69	5.04E-09	5.49E-11	2.32E-04	1.96E-16
	**ERVMER34-1**	-1.71	-0.89	-1.05	-0.53	3.64E-11	1.93E-11	7.13E-08	2.55E-05
	**FBLN2**	-1.14	-0.53	-0.70	-0.55	2.19E-06	5.11E-07	4.20E-05	1.77E-08
	**IGFBP2**	-1.08	-0.60	-0.55	-0.49	8.69E-03	5.38E-09	3.45E-03	1.58E-04
	**IGFBP4**	-1.70	-0.67	-0.70	-0.30	2.19E-06	8.01E-08	9.67E-05	4.30E-03
	**NID1**	-1.37	-0.61	-0.82	-0.53	1.95E-07	1.29E-07	1.08E-04	1.49E-05
	**NPTX2**	-2.02	-2.73	-0.90	-0.93	7.42E-07	3.82E-16	1.85E-07	3.48E-18
	**NTS**	-2.07	-1.81	-2.79	-1.60	1.56E-08	3.35E-09	3.18E-48	3.46E-20
	**PLTP**	-0.58	-0.52	-0.54	-0.43	9.59E-04	1.44E-06	4.07E-03	1.59E-06
	**PODXL2**	-3.19	-1.38	-0.49	-0.40	6.52E-03	3.25E-03	1.64E-02	6.06E-04
	**SEMA3A**	-1.41	-1.11	-0.56	-0.47	3.09E-07	9.58E-14	1.11E-02	2.25E-03
	**SEMA3F**	-1.06	-0.60	-0.44	-0.47	8.11E-07	9.54E-07	3.14E-02	7.86E-06
	**SFRP2**	-1.87	-0.84	-1.45	-0.81	5.91E-11	1.79E-09	3.85E-16	4.84E-21
	**STC2**	-1.23	-0.79	-1.04	-0.76	2.50E-04	2.84E-06	2.65E-06	5.19E-14
	**THY1**	-0.86	-0.83	-1.66	-1.46	9.11E-05	1.99E-11	1.39E-28	3.02E-50
	**TNFRSF8**	-4.17	-0.83	-0.70	-0.52	5.65E-03	7.47E-13	2.69E-05	8.61E-06
	**WFDC2**	-1.35	-0.83	-0.97	-0.84	4.40E-06	5.29E-12	1.09E-08	2.59E-12

### Confirmation of marker proteins by Western blotting

**Fig 6:**
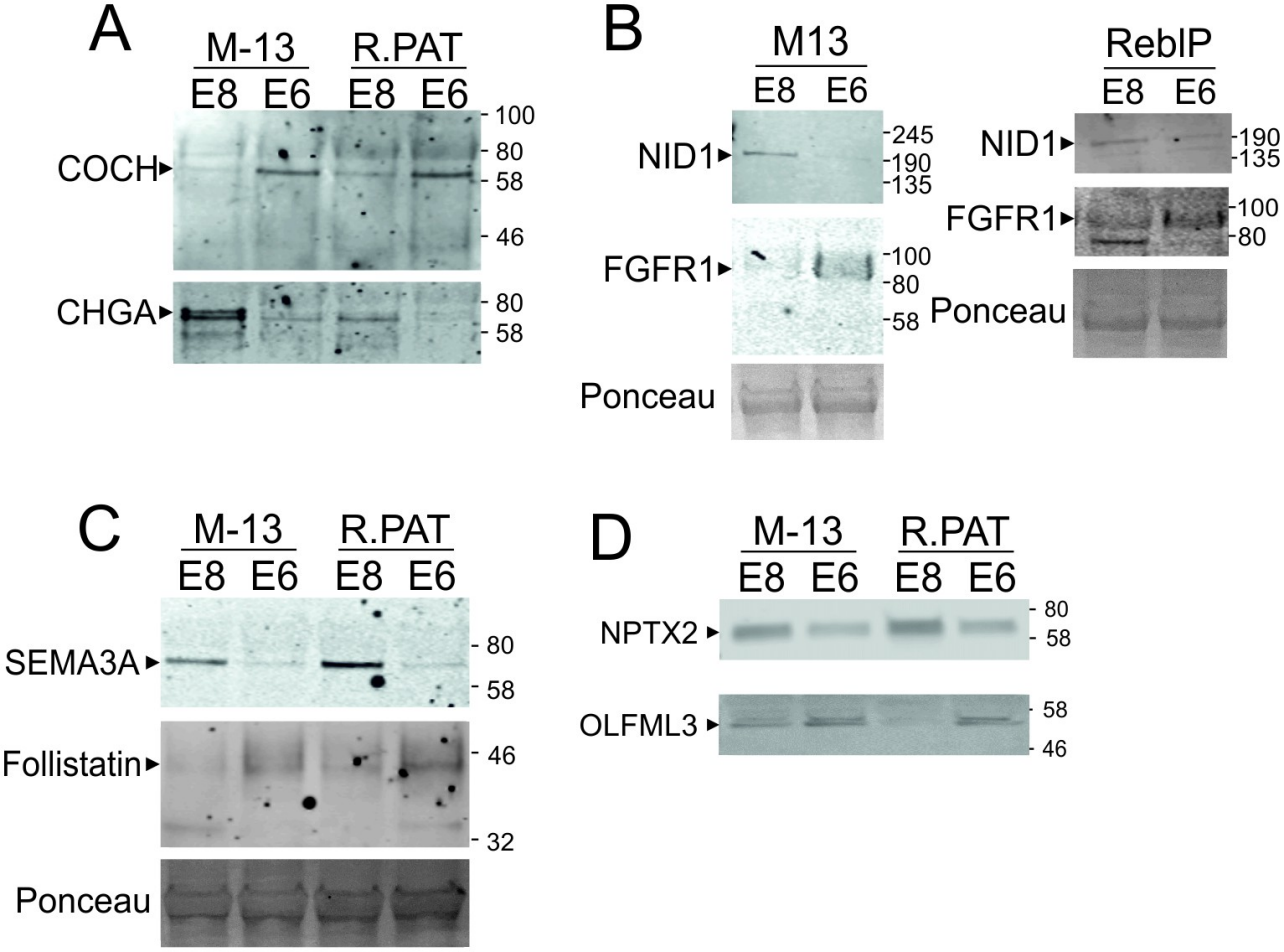
Western blot confirmation of marker proteins. Equal quantities of protein from media supernatant from cells cultured as described in Fig 1 were run on Western blot and probed with antibodies for an E8-enriched marker protein (CHGA, NID1, SEMA3 or NPTX3) and an E6-enriched marker protein (COCH, FGFR1, Follistatin or OLFML3). Membranes were imaged using LICOR Odyssey system.

Based upon the strength of these markers in the LC-MS/MS data, equal quantities of protein from conditioned media samples (collected as described in Fig 1A) were probed by Western blot for the relative abundances of these marker proteins where suitable antibodies were available (Fig 6). In the absence of a conditioned medium loading control, each E8-marker protein was probed for on the same membrane with a corresponding E6-marker protein to confirm the utility and validity of the internal normalisation method. The Western blot data was consistent with MS results: NID1, SEMA3A, CHGA and NPTX2 bands were stronger in E8 than E6 while COCH, FGFR1, FST and OLFML3 bands were stronger in E6 medium.

### Recovery of protein-pair ratio abundances after 48 hours in E6 medium

**Fig 7:**
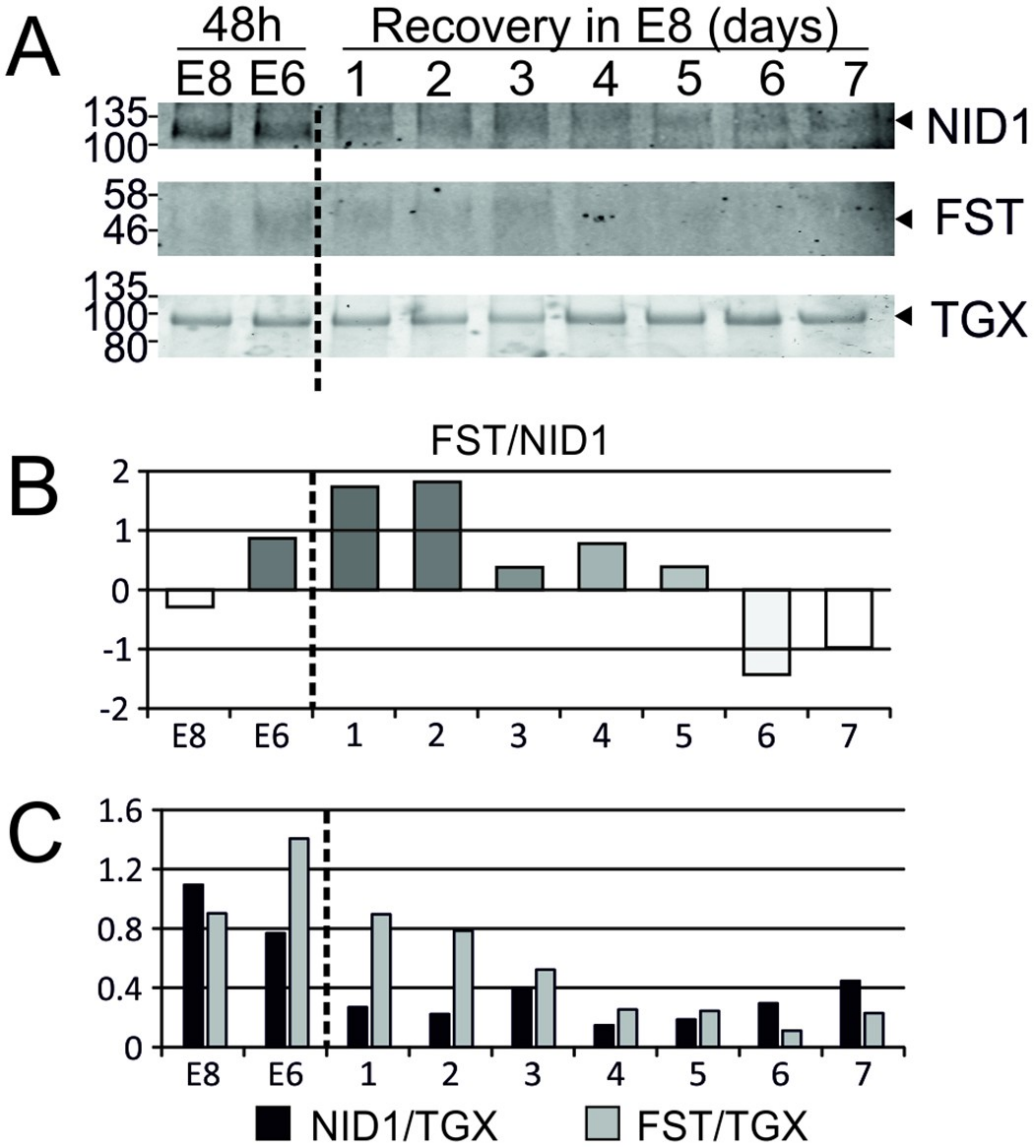
Re-establishment of relative marker abundances after a 7-day rescue. For WIGW2 cells cultured as described in (Fig 1), but after media collection at 48 hours. E6 cultured cells were passaged and returned to E8 for 7 days with media collection every 24 hours. A) TGX Stain-free gels were loaded with 20ug of protein and imaged before transfer to membrane—the most prominent gel band is shown as a demonstration of equal loading. The membrane was probed with antibodies FST & NID1. B) Quantification shows the Log2-transformed ratio of FST/NID1 band intensity. C) Individual FST & NID1 band intensity, normalised to most prominent TGX band. Abundances calculated from densitometry in ImageJ. Membranes were imaged using the LICOR Odyssey system.

An important component of an assay for loss of pluripotency is the ability to detect a degradation of culture quality while the cells are recoverable. Therefore, we repeated the experiment, but replated cells into E8 medium for 7 days after the 2 days in E6. In cell lines WIGW2 (Fig 7) and Man1 (S4 Fig), we show that the change in the E6/E8 marker protein abundance ratio caused by 48 hours in E6 is reversed after 6 days of culture in E8, both in WIGW2 hiPSCs and Man-1 hESCs, using FST/NID1 and OLFML3/NID1 marker pairs respectively.

Moreover, cells cultured for 48 hours in E6 and recovered for 7 days in E8 media can still form teratomas (S5 Fig) demonstrating differentiation to tissues of all three germ layers. This suggests not only that the pluripotency of these cells can be fully recovered after 48h in E6, but also that our marker proteins can robustly track this recovery. That the changes we observe are not simply molecular markers of the immediate loss of growth factor is supported by the fact that the proteins take several days to return to their baseline, rather than returning within 12–24 after the FGF2 and TGFβ1 are restored. Thus, at the time early loss of pluripotency is recognised by these early marker changes, cultures are still plastic and can recover.

### Assessment in TeSR1 and mesodermal differentiation medium

In order to further validate that some of these secretome markers could be predictive with cells cultured in other commonly used media we grew Man13 in the pluripotent stem cell culture medium, TesR1, (S6 Fig) and chondrogenic differentiation medium [25]. TesR1 medium has high HSA which interferes with Western blotting and so this had to be removed before running the gel (see Methods). Three separate 5ml medium samples of TESR1 were concentrated to contain 50μg, 100μg and 200μg of protein in total (after HSA removal) and quantified using a BCA assay prior to western blotting. NPX2 was observed in all lanes in the TeSR1 medium after HSA extraction (S6B Fig). A clear Follistatin band was detected strongly in the E6 medium using only 20μg protein in comparison to, and stronger than, 50μg protein from the TESR1 pluripotency medium. This confirms the enhancement of Follistatin on transfer of stem cells to a pro-differentiation medium (S6A Fig). Similarly, when cells were transferred to a chondrogenic differentiation medium for 48h after culture in TeSR1, there was an increase in Follistatin and decrease of NPTX2 (S6B Fig). NID1 was detected in TeSR1 after hESC culture but only at 200ug total protein and it is likely that this may be partly removed with the HSA.

## Discussion

We first investigated changes in commonly employed pluripotency associated proteins after only 48 hours following FGF2/TGFβ removal from the stem cell culture medium, confirming that neither immunofluorescence nor flow cytometry for these markers could confidently detect pluripotency loss at this time. Although it is a pluripotency-associated marker, SOX2 increased slightly on transfer to E6 lacking FGF2 and TGFβ. This is to be expected as SOX2 expression has previously been found to increase upon SMAD2/3 inhibition and FGF2 deprivation [26]. Moreover, increases in SOX2 in hPSCs can initiate differentiation [27, 28] and SOX2 has an important role in specification and differentiation of PSCs towards the neurectoderm lineage [29–31].

In this study we identified a series of proteins which function as secreted, early marker proteins for incipient loss of the pluripotent stem cell state in culture. To examine if the identified protein changes indicated a change in protein synthesis, we also investigated gene transcription and found that the proteome changes were indeed also generally reflected in changes in the respective gene transcripts. From this list of marker proteins (Table 1) a shortlist of eight proteins was identified for further analysis by Western blotting.

### Selected marker protein biology

Although the cellular proteomes of both hESC and hiPSC lines have been investigated by a variety of mass spectrometry techniques in the past [7–10] the protein secretome of these cells has been under studied [11]. Using a combination of LC-MS/MS and bioinformatic analysis with confirmation by Western blotting, we identified several markers in the secretome indicative of healthy pluripotent stem cells and early incipient pluripotency loss. Moreover, we have also shown that cells returned to E8 after 48h in E6 show recovery and exhibit secretome marker proteins correlating with the pluripotent state again.

The transmembrane FGF2 receptor FGFR1 was one of the most highly represented secreted markers identified to increase upon growth factor withdrawal. There are several possibilities to explain the increased incidence of FGFR1 in the conditioned E6 medium. Firstly, FGFR1 is known to be released into the extracellular space by cleavage in its membrane-adjacent extracellular domain by MMP2 [32]. As FGFR1 proteins are endocytosed by the cell upon substrate binding and degraded in lysosomes [33–35], it is likely that decreased FGF2 abundance will result in an increased surface abundance of FGFR1, and a corresponding increase in the amount of FGFR1 which is cleaved by surface metalloproteinases. Indeed, a majority of the FGFR1 peptides identified as significantly increased in E6 in our proteomics study were N-terminal to the proposed MMP2 cleavage site. As it has previously been reported that a secreted form of the extracellular region of FGFR1 inhibits FGF2 activity [36] it is possible that the FGFR1 accumulation in FGF2 deficient media may further inhibit residual FGF2 signalling.

Indeed, the biological functions of other E6-enriched protein biomarkers are consistent with a hypothesis that cells triggered towards differentiation release factors into the medium that bind and modulate growth factors, sequestering pluripotency promoting factors such as FGF2 and TGFβ 1, or stabilising other growth factors, to regulate their local availability. This will generate a feed-forward amplification of the original pluripotency dissolution signal which further highlights the need for early warning of incipient pluripotency loss. Follistatin is a well-known stem cell differentiation factor, and TGFβ inhibitor, functioning through the binding and neutralisation of TGFβ superfamily members [14]. Similarly, OLFML3, also enriched in E6, has been shown to bind to and stabilise BMP4, enhancing SMAD1/5/8 signalling in endothelial cells [37]. As BMP4 initiates differentiation of human embryonic stem cells to trophoblast in the absence of FGF [38–40] and to mesoderm and chondrocytes [41, 42], it could be concluded that after 48 hours of FGF2/TGFβ loss, these cells are secreting factors to facilitate specification to differentiated lineages [43]. In murine ES cells COCH has been reported to be expressed in response to BMP4 signalling [44], in our study COCH was enhanced in E6 medium possibly reflecting the loss of FGF which is known to suppress BMP signalling in hESCs [12]. The molecular differences in the pluripotent ESC state between human and murine reflect an earlier naïve murine ESC phenotype and later primed human ESC phenotype [45] and are supported by the wealth of data on the differences in signalling required for murine and human stem cell maintenance [12, 13, 46, 47]. Thus, BMP signalling has different effects in hESCs and murine ESCs, being active in stem cell maintenance in the latter [39, 47]. Since BMP signalling is strongly associated with differentiation to mesodermal and trophectodermal lineages in hESCs [38, 41, 48–50], this would be in keeping with a correlation between targets such as COCH down stream of BMP and the dissolution of pluripotency precipitating incipient lineage differentiation.

SEMA3A, a neuronal signalling protein involved in axon guidance, is highly enriched in the E8 condition by both secretome proteomics and RNAseq, whilst its transmembrane receptor, NRP1 is highly enriched (q<0.01) in the E6 transcriptome [51–53]. NRP1 also binds to both FGF2 and TGFβ and modulates their signalling. In HUVEC cells, increased NRP1 expression was demonstrated to suppress Smad2/3 activation upon TGFβ stimulation, and expression of SMAD target genes in response to TGFβ was increased in NRP1 deficient HUVECs [54], a relationship which has been replicated *in vivo* in murine endothelial cells [55]. NRP1 is a highly promiscuous receptor, and ligands compete for NRP1 binding such that competitive inhibition between ligands is common [56]. So NRP1 may also play a role in the modulating TGFβ and other growth factor activity in the transition from pluripotent stem cells to early differentiation.

CHGA is a precursor protein for several neuroendocrine signalling proteins. Peptides from across the whole CHGA molecule were identified as being highly enriched in E8 conditioned medium over those in E6 conditioned medium. Generally speaking, CHGA drives formation and release of secretory granules [57, 58], and its decreased abundance in stem cells during pluripotency dissolution could reflect a reduction in overall secretion. This would be consistent with the greater number of secreted proteins identified in E8 conditioned medium compared with in E6 though further data is needed to confirm this association.

NID1 is a secreted glycoprotein with two principal protein-binding domains (G2 and G3), separated by a flexible chain [59]. By domain-specific binding of different components of the basement membrane, NID1 stabilises the basement membrane, cross-linking its multiple components [60, 61]. NID1 is abundant in locations where there is a requirement for additional resilience against mechanical stress, and conversely, less abundant where more flexibility is required and tissue disposition is not fixed [62] for instance being actively degraded during basement membrane disassembly [63]. It has been demonstrated by proteomic analysis to be expressed at significant amounts by hESCs [11, 64]. Here we observed a dramatic reduction in the abundance of NID1 in E6 medium compared with E8. As NID1 has a stabilising effect on the basement membrane, it is likely that its abundance is decreased prior to the epithelial-mesenchymal transition inherent in the progression from a pluripotent stem cell to an early progenitor. This will involve the disassembly of several extracellular matrix complexes: in ovarian cancer cells NID1 plays a key role in this EMT transition [65].

NPTX2 binds to α-amino-3-hydroxy-5-methyl-4-isoxazolepropionic acid (AMPA) receptor subunit Glutamate receptor 4 (GRIA4) in neuronal tissues [66] and has a presynaptic role in Pyramidal neurons with loss/loss of function associated with Alzheimer’s disease [67] and Schizophrenia [68]. It is noteworthy that the expression of *GRIA4* is also much higher in E8 than E6 (Supplementary RNAseq data) as for NPTX2, suggesting some as-yet unidentified function for this ligand-receptor pair in hPSCs. NPTX2 is also a circulating biomarker of pancreatic cancer [69]. In glioblastoma NPTX2 has been shown to inhibit AKT which in turn represses NFKB activity [70]. There is evidence for AKT and NFkβ involvement in pluripotent stem cell maintenance [71] with PI3k/AKT being involved in switching Smad2/3 signalling between a pluripotent self-renewal and differentiation role through controlling ERK and GSK3β signalling [72].

Although we are unclear about the precise role of some of our pluripotent stem cell and early pluripotency dissolution secretome markers in the switch from self-renewal to early differentiation, we have robust evidence of their utility to predict the switch, suggesting that these marker proteins will be of commercial interest and in generation of cell therapies, where efficiency, robust and reliable cell output and cost effectiveness are paramount.

### Development of paired-ratio method

A caveat of these analyses, common to all secretome studies, is the lack of a suitable normalisation standard against which to compare proteins to robustly identify changes between conditions. Typically, ‘omics’ studies normalise to total protein or mRNA, or to house-keeping genes or proteins such as GAPDH or β-actin. These controls allow for reliable, reproducible comparisons across conditions or experiments. When analysing the secreted complement of the cell proteome, however, no such house-keeping protein exists, and the overall protein abundance between conditions is highly susceptible to variability due to the presence and concentration of medium proteins, the density of cells, differing cell viability, cytoplasmic leakage, and a host of other factors. In this study, we endeavoured to limit the effect of these variables, and further sought to identify marker proteins which could be multiplexed against each other to provide ratios which are much more discriminatory between the experimental conditions. Regardless of other changes in the cell secretome, the relative abundances of these proteins should remain relatively constant within an individual genetic background, culture method, and cell state. As such, once baseline ratios are identified under control conditions, it is expected that these ratios would provide a rapid, quantitative warning of imminent pluripotency loss in cultured hPSCs, above and beyond any current method in terms of sensitivity, affordability, and rapidity of timescale.

In summary we have shown that there are several proteins in the secretome of hPSCs that are rapidly decreased or increased during dissolution of pluripotency. By employing a matrix of four of each such species, robust ratios of proteins could be generated which were indicative of stable pluripotency or its incipient loss. Additionally, other proteins were identified by MS/MS and will make excellent candidate biomarkers and provide biological interest for follow-up analyses. Such proteins will be invaluable as markers allowing intermittent or continuous culture monitoring using rapid turnaround techniques such as ELISA or high-throughput targeted MS, during the scale up and manufacture of hPSCs for cell therapy or for their use in pharmaceutical drug development and toxicity testing. For commercial use, in-line MS monitoring of media samples for specific proteins and their ratios would be possible to give early warning of loss of stem cell status provided that MS quantitation is a reliable output. The development of aptamers or nanobodies is exciting as they can be used with a variety of biosensor technologies. Optical sensors such as multi-parametric surface plasmon resonance, and biolayer interferometry may be sensitive enough to accurately quantify the ratio of biomarkers directly but electrochemical detectors can enhance detection limits. They can be developed relatively cheaply giving a cost-effective means of monitoring with such markers. These devices will be equally valuable in the research laboratory, allowing rapid evaluation of culture status without loss of cell product.

## Materials and methods

### Cell culture

Cells were maintained in Essential 8 (E8) medium (Life Technologies) on Vitronectin-N (Life Technologies) and passaged using TrypLE dissociation reagent (Life Technologies). Prior to sample collection for secretome analysis, cells were plated in E8 medium, supplemented for 24 hours with 10μM ROCK inhibitor, at a density of 20,000 (Rebl.PAT) or 25,000 (Man-13) cells per cm^2^ in 75cm^2^ culture flasks (Corning). Twenty-four hours after plating (experimental time 0), cells were either fed with fresh E8 medium, or transferred to Essential 6 (E6) medium (Life Technologies). Cells were fed at 48 and 72 hours (experimental time 24h and 48h respectively) after plating and at these time-points medium was collected, spun at 1,000 x g at -9°C for 5 minutes then aliquoted into lo-bind 2ml centrifuge tubes (Eppendorf) and snap frozen in liquid nitrogen. At 72 hours after plating (experimental time 48h), cells were dissociated with TrypLE, spun at 1,000 x g in PBS for 5 minutes, the supernatant removed, and the pellet snap frozen in liquid nitrogen. Viability assays were conducted at medium harvest using Apotox-Glo assay (Promega) [73] or with the NucleoCounter NC-200 and Via-1 cassettes (Chemometec) according to manufacturers’ instructions.

A subset of the cells at this time-point were also collected and processed for RNA-Seq analysis. Five biological replicates were conducted for Rebl.PAT, each of which was composed of three technical replicates, and all samples were analysed by LC-MS/MS in a single run. To ensure quantitative reproducibility of the marker proteins identified across multiple analyses, four Man-13 biological replicates were collected, and these were analysed in two independent LC-MS/MS runs consisting of two biological replicates each. Some Man13 cells were also cultured in TeSR1 medium (Stem Cell Technologies) and then transferred to E6, or chondrogenic medium (DMEM:F12, 2mM L-glutamine, 1% (vol/vol) ITS, 1% (vol/vol) nonessential amino acids, 2% (vol/vol) B27, 90μM β-mercaptoethanol (all Life Technologies) + 20ng/ml BMP2 (R&D systems) 10ng/ml ActivinA (Peprotech, 20ng/ml FGF2 (Peprotech), 3μM CHIR99021 (Tocris), 100μM Pik90 (Selleckchem), modified from Wang et al 2019 [25].

An important component of an assay for decreased PSC culture quality is the ability to detect incipient pluripotency loss while the cells are still recoverable. The experiments above were repeated but after 2 days in E6, cells were restored to E8 medium for 7 days to assess whether full pluripotency could be recovered at the time that protein ratios detected early changes. Medium was refreshed every 24hrs with samples of conditioned medium being taken for analysis by Western Blot every 48 hours.

### Immunochemical staining of pluripotency markers

Cells were seeded at a density of 20,000 (Rebl.PAT) or 25,000 (Man-13) cells per cm^2^ in a 24 well plate and treated identically to samples prepared for proteomic and RNA-Seq analysis (Fig 1A). Cell surface markers and transcription factors characteristic of pluripotent hESCs were detected using immunofluorescence. The cells were fixed with 4% paraformaldehyde and incubated with antibodies against stage specific embryonic antigens SSEA-4, SSEA-1 (R&D Systems), TRA-1–60, TRA-1-81 (Abcam) and transcription factors SOX2, NANOG (Cell signalling Technologies), and OCT-4 (BD Biosciences) at 4°C overnight. Secondary antibodies (Life Technologies), specific for the species and isotype of the primary antibody, conjugated to Alexafluors 488 or 594 were used for detection using a BX51 microscope (Olympus, Hertfordshire, UK) equipped with a Q-Imaging camera (Micro Imaging Applications Group, Inc, Buckinghamshire, UK). Image processing was done with the aid of Q-Capture Pro software package (Micro Imaging Applications Group, Inc).

### Gene expression analysis

Total RNA was extracted using mirVana^™^ miRNA Isolation Kit (Life Technologies), reverse transcribed using M-MLV reverse transcriptase (Promega) and candidate genes expression (normalised to GAPDH) assessed using SYBR Green PCR Master Mix (Applied Biosystems) with an ABI PRISM 7500 Real Time System (Applied Biosystems). Primers for rtPCR were as follows: GAPDH -FW (5’- ATGGGGAAGGTGAAGGTCG), GAPDH -RV (3’- TAAAAGCAGCCCTGGTGACC), Sox2 -FW (5’- AACCAGCGCATGGACAGTTAC), Sox2 -RV (3’- TGGTCCTGCATCATGCTGTAG), Oct4 -FW (5’- AGACCATCTGCCGCTTTGAG), Oct4 -RV (3’-), NANOG -FW (5’- GGCTCTGTTTTGCTATATCCCCTAA) and NANOG -RV (3’- CATTACGATGCAGCAAATACAAGA).

### Flow cytometry

Cells were collected at the time of plating, and at the 48-hour time-point for each experiment, as well as at the 24-hour time-point for a subset of experiments. All flow cytometry analyses were performed on a BD LSRFortessa^™^ cell analyser (Becton-Dickinson, San Jose, CA). Cells were dissociated using TrypLE Express (Life Technologies), fixed in the dark for 7 minutes at room temperature in 4% paraformaldehyde, blocked for thirty minutes to two hours in 5% FBS and were permeabilised for intracellular staining in ice-cold 70% methanol. Species and fluorochrome-matched isotype controls were used for each antibody to control for non-specific binding.

Cells were incubated with directly conjugated primary antibodies for 30 minutes at room temperature, at the following dilutions; Phycoerythrin-conjugated mouse anti-TRA 1–81 (Ebioscience 12-8883-80), 1:300; Phycoerythrin-conjugated mouse isotype control (Santa-Cruz, SC2870) 1:75; Alexa 488-conjugated Mouse anti-Nanog (BD Bioscience, BD560791), 1:10; Alexa 488-conjugated mouse isotype control (BD Bioscience, BD557702) 1:100; Alexa 647-conjugated mouse anti-Sox2 (BD bioscience, BD56139) 1:80; Alexa 488-conjugated mouse anti-OCT4 (R&D systems, IC17591G) 1:50; Alexa 647-conjugated mouse isotype control (BD Bioscience, BD557714) 1:80. Staining for TRA-1-60 was performed using a non-directly conjugated antibody. Cells were incubated with the primary antibody mouse anti-TRA-1-60 (Invitrogen, MA1-023, dilution 1:400), followed by incubation with Alexa 488-conjugated goat-anti-mouse IgM secondary antibody (Invitrogen, A-21042, dilution 1:2500). Flow cytometry data was collected and preliminarily processed in FACSDIVA^™^ software followed by analysis and figure-preparation in FlowJo (both Becton-Dickinson, San Jose, CA).

### Proteomics sample preparation

Five biological repeats each consisting of three technical replicates of Rebl.PAT cells were performed, and all analysed together by label-free mass-spec (Fig 2A). Man-13 samples were analysed by LC-MS/MS in two batches of two biological repeats (each consisting of three technical replicates) to ensure that any identified biomarkers would be resilient to run variation (Fig 2A). A subset of the cells was also collected and processed for RNA-Seq analysis at the 48-hour time-point.

Media samples were digested using a modified Filter-assisted Sample Preparation (FASP) method [74] with the following modification: Equal volumes of conditioned media were concentrated to approximately 50 μL with Microcon—10 kDa centrifugal filter units (Merck Millipore) at a speed of 14,000 x g. This was then washed and centrifuging three times with the addition of 100 mM phosphate buffer pH 7.4 before the proteins were reconstituted in 50 μL of phosphate buffer. The protein concentration was determined using a Millipore Direct Detect^®^ spectrometer and 50 μg (Man-13) or 25 μg (Rebl.PAT) of protein was added to a fresh 10 kDa filter tube with reduction, alkylation and digestion occurring using the filter tubes. After digestion peptides were collected by centrifugation and the samples were desalted with OLIGO^™^ R3 reversed-phase media [75] on a microplate system dried to completion and reconstituted just before analysis in 5% acetonitrile and 0.1% formic acid.

### LC-MS/MS

Digested samples were analysed by LC-MS/MS using an UltiMate 3000 Rapid Separation LC (RSLC, Dionex Corporation, Sunnyvale, CA) coupled to an Orbitrap Elite (Thermo Fisher Scientific, Waltham, MA) mass spectrometer. Peptide mixtures were separated using a multistep gradient from 95% A (0.1% FA in water) and 5% B (0.1% FA in acetonitrile) to 7% B at 1 min, 18% B at 35 min, 27% B in 43 min and 60% B at 44 min at 300 nL min-1, using a 75 mm x 250 μm i.d. 1.7 μM CSH C18, analytical column (Waters). Positive Polarity source voltage (ESI) voltage was 1.7Kv. Peptides were selected for fragmentation automatically by data dependant and the top 9 peaks were selected MS2 analysis. The total run time for each sample was 90 minutes.

### Identification and quantification of peptides

The acquired MS data was analysed using Progenesis LC-MS (v4.1, Nonlinear Dynamics). The retention times in each sample were aligned using one LC-MS run as a reference, then the “Automatic Alignment” algorithm was used to create maximal overlay of the two-dimensional feature maps. Where necessary a minimal amount of manual adjustment was employed to increase alignment score to above 80%. Features with charges ≥ +5 were masked and excluded from further analyses, as were features with less than 3 isotope peaks. The resulting peak lists were searched against the SwissProt (release 2016–04) and Trembl (release 2016–04) databases using Mascot v2.5.1, (Matrix Science). Search parameters included a precursor tolerance of 5 ppm and a fragment tolerance of 0.6 Da. Enzyme specificity was set to trypsin and one missed cleavage was allowed. Carbamidomethyl modification of cysteine was set as a fixed modification while methionine oxidation was set to variable. The Mascot results were imported into Progenesis LC-MS for annotation of peptide peaks. The Proteomic datasets generated in this study have been deposited to the ProteomeXchange Consortium via the PRIDE partner repository with the dataset identifiers PXD045525 & PXD045347.

### Proteomics data processing

SwissProt and Trembl IDs were used to align data between independent proteomics experiments. Normalised protein abundance data from Progenesis were log2 transformed to improve the normality of distribution. All proteins used for statistical analysis had one or more unique peptides, and a Progenesis confidence score of 30 or more in their MS experiment. Statistical significance was calculated using linear regression models, fitted independently for each protein using the lm package in R and the formula *ProteinAbundance ~ Media + BiologicalReplicate*. The p-values from these models were then adjusted for multiple comparisons using the Benjamini & Hochberg (q) method.

Peptide sequences for all proteins were uploaded to the SignalP 5.0 server [20] to identify the proportion of proteins bearing a classical secretion signal-peptide sequence. Non-classical secretion was identified by uploading peptide sequences to the SecretomeP 2.0 server [21]; and the recommended cut-off score of 0.6 was used to quantify the proportion of proteins which were likely to be secreted through non-classical pathways. Protein GO-term associations were obtained by from Uniprot, or by use of WebGestalt’s GO-Slim output [76].

### RNASeq data analysis

BAM files were aligned to the human genome using BowTie [77]. In Linux, BAMs were sorted in the command interface using samtools [78], and the number of read counts per gene was obtained using featureCounts [79] (supplemental script 1). For each cell line, a count matrix table was constructed from all the counted BAMs.

The following analysis was conducted in R [80]. Genes with less than 10 reads in all three replicates were filtered out. Counts were normalised using TMM [81] and normalisation factors for each cell line generated. Subsets of filtered counts and normalisation factors were created to represent samples relating to loss of pluripotency. Differential analysis was performed using DESeq2 [82]. Statistically significant differentially expressed genes are defined using FDR corrected p-value <0.05.

### Western blotting

Medium from a variety of cell lines, including WIGW2, Man-1, Man-7 and H9 cells in addition to MAN-13 and Rebl.PAT, was collected as described in Fig 1A, and 3ml of medium was concentrated to 50–100μl in Microcon– 10 kDa centrifugal filter units (Merck Millipore). Samples were run on 10% Bis-Tris gels (Thermo #NW00100BOX), or TGX Stain-Free gels (BIO RAD #4568021) on a Mini Gel Tank (Thermo #A25977) using MES SDS Running Buffer (Thermo #B0002) alongside broad range markers (11-245KDa, NEB #P7712S). Each lane contained 20 μg of protein, heated in Pierce lane marker reducing buffer (Thermo #39000) at 95°C for 10 minutes. TGX Stain-Free gels were activated prior to membrane transfer (45 seconds, UV Light Chemidoc MP imaging system). Gels were transferred using the IBlot2 Gel Transfer device (Thermo #IB21001), using iBlot 2 Transfer stacks (nitrocellulose membrane, Thermo #IB23001).Cells were analysed by immunoblotting using the following antibodies: 1/200 Mouse anti-Chromogranin A (Novus Biologicalis, NBP2-44774); 1/100 Rabbit anti-COCH (Abcam, ab170266); 1/500 mouse anti-FGFR1 (R&D systems, MAB658); 1/1000 rabbit anti-Follistatin (Abcam, ab157471); 1/1000 rabbit anti-Entactin/NID1 (Abcam, ab133686); 1:1000 rabbit anti-Neuronal Pentaxtrin 2 (Abcam, ab191563); 1/1000 rabbit anti-Neurotensin (Abcam, ab172114); 1/300 rabbit anti-Olfactomedin-like 3 (Abcam, ab111712); 1/500 mouse anti-Semaphorin 3A (R&D, MAB1250); 1/500 mouse anti-Secreted Frizzled Related Protein 2 (R&D, MAB6838). Secondary antibody staining was performed either using 1/20,000 IRDye 800CW Goat anti-mouse, or 1/20,000 IRDye 680RD Donkey anti-Rabbit (P/N 925–32210 and 925–68071 respectively, both from LI-COR). These fluorescent secondary antibodies were imaged using the Odyssey CLx imaging system (LI-COR) typically in pairs of rabbit and mouse antibodies together. Image brightness/contrast adjustment and densitometry quantification was all performed in ImageJ (NIH).

### Protein extraction method for Western blotting

Media was concentrated using 10KDa Micron filter spin columns (Millipore), centrifuged at 14,000xg for 40mins at 4C, followed by 2 washes with PBS (Life Tech) and spinning at 14,000xg for 15mins at 4C. Protein was resuspended to a total volume of 200μl with 1x PBS. Protein concentration was determined using a BCA Assay kit (Pierce). Concentrated proteins were mixed and vortexed for 2 mins with 1% Trichloroacetic acid- Isopropanol (TCA-IPA) solution (1:10 ratio) and centrifuged 1500xg for 5mins at 5C. Pellets were washed twice with methanol, centrifuged at 800xg for 2mins and resuspended in 6x SDS-PAGE sample buffer, boiled at 96C 10mins and loaded onto an SDS gel for analysis.

### Teratoma formation

Teratoma generation was by subcutaneous injection into Scid Beige mice of approximately 1 million pelleted cells in Matrigel^™^ [83]. Animals were killed within 11 weeks of injection; skin biopsies fixed in 4% paraformaldehyde and embedded in paraffin wax for histological analysis. Some sections were stained with antibodies to early endoderm (GATA6- CST 5851 1:1600) neurectoderm (Beta3 tubulin R&D Systems MAB1195 1:200) or smooth muscle actin in mesoderm (alpha SMA R&D Systems MAB1420 1:200) followed by incubation with biotinylated secondary antibodies (Vector Labs biotinylated secondaries, anti-mouse, BP-9200 and anti-rabbit IgG BP-9100) and developed using the ABC peroxidase kit (Vector labs PK-6100). Slides were imaged using the 3D Histec Pannoramic250 slide scanner and assessed using CaseViewer with Histoquant licence.

All mice were housed and handled in accordance with applicable Home Office guidelines. Local ethical approval was granted, and the work was done under UK home office licence 70/7838. Surgery was performed under isoflurane anaesthesia, and all efforts were made to minimize suffering.

## Supporting information

S1 Fig A) Immunocytochemistry of Man-13 cells cultured in E8 (control) or after 48 hours of culture in E6. B) Flow cytometry of Rebl.PAT and Man-13 cells cultured in E8, or after 48 hours of culture in E6. For Rebl.PAT cells, extra flasks were cultured alongside experimental flasks, and these were used for observation of cell state at the 24-hour time-point (shown for Rebl.PAT). C) QRTPCR of Man-13 and Rebl.PAT cells cultured in parallel to those used for medium collection, either maintained in E8, or after being incubated for 48 hours in E6. QRT-PCR for pluripotency associated markers Oct4 Sox2 and Nanog relative to GAPDH.
(TIF)

S2 Fig Viability of cells after 48 hours of culture in E6. At the 48-hour timepoint (Fig 1), viability of E8 and E6 cultured cells was assessed using Via-1 cassettes (Chemometec). All three flasks of cells for each condition were assayed, and no significant change between the conditions was observed in any experiment.
(TIF)

S3 Fig Principal Component Analysis (PCA) of all secreted proteomics samples. PC1 and PC3 are shown to demonstrate firstly the necessity of controlling for MS run in the statistical analysis (PC1, 67.2% of variance), and secondly that despite the other sources of variance, the experimental conditions are still clearly separated at PC3.
(TIF)

S4 Fig Re-establishment of relative marker abundances in Man-1 after a 7-day rescue. Man-1 cells were cultured under the same conditions as those in Fig 7, however the Western blot membrane was probed with OLFML3 & NID1 antibodies. Quantification shows the Log2 ratio of E6/E8 marker abundance (OLDML3/NID1). Densitometry was performed in ImageJ. Membranes were imaged using the LICOR Odyssey system.
(TIF)

S5 Fig Paraffin sections (7μm) of teratomas generated by subcutaneous implantation of human pluripotent stem cells cultured in in E8 (Man13) or after 2 days in E6 Man13 and Rebl.PAT) stained for morphological and protein markers of the 3 germ layers. Paraffin sections were stained with Haematoxalin and eosin or with antibodies to early endoderm (GATA6) neurectoderm (Beta3 tubulin) or smooth muscle actin in mesoderm (alpha SMA) followed by a peroxidase labelled secondary antibody. Scale bar 100um.
(TIF)

S6 Fig A) NPTX2 / FST Ratio of concentrated TeSR1 pluripotency media from cells grown for 48hrs, and medium subjected to HSA removal using 1%TCA-IPA or without HSA removal. B) NPTX2 / FST Ratio in media concentrated from cells grown in TeSR1 pluripotency medium versus mesodermal (stage1 chondrogenesis) differentiation medium showing that the change in protein ratios for NTPTX2 and Follistatin holds in both experiments if HSA is removed.
(TIF)

S1 Raw images (PDF)
