# Correction: A secreted proteomic footprint for stem cell pluripotency

**DOI:** 10.1371/journal.pone.0317156

**Published:** 2025-01-02

**Authors:** Philip A. Lewis, Edina Silajdžić, Helen Smith, Nicola Bates, Christopher A. Smith, Fabrizio E. Mancini, David Knight, Chris Denning, Daniel R. Brison, Susan J. Kimber

The datapoints, axes and trend-line of Rebl.PAT are missing in Fig 3. Please see the correct Fig 3 here.

**Fig 3:**
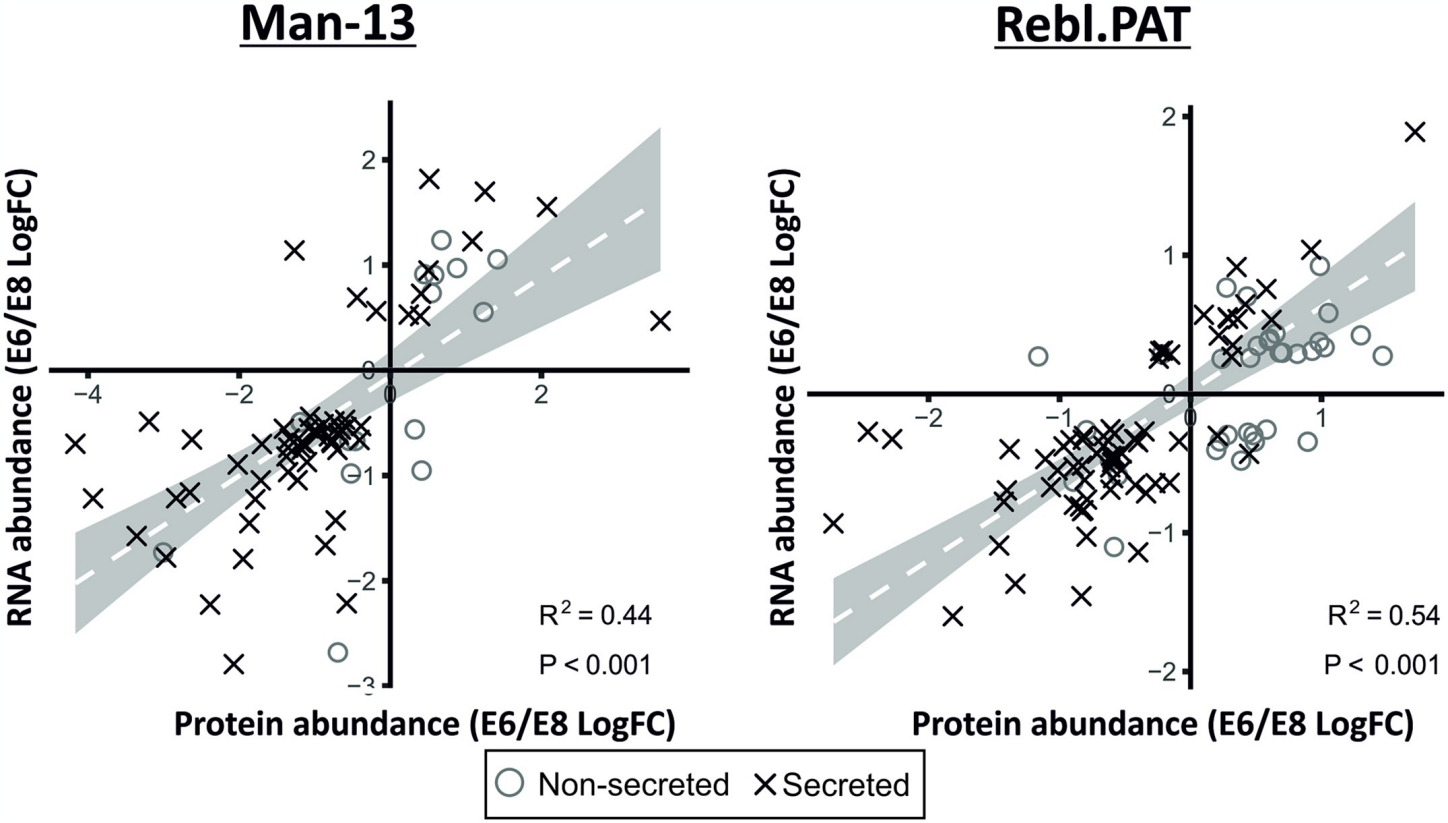
Correlation between E6 /E8 log fold-changes between mRNA and protein data. Protein data was paired to RNA-Seq data using Entrez IDs retrieved from Uniprot and Bioconductor [22–24]. These data show a correlation between the changes observed in significantly changed (q<0.05) secreted proteins and the corresponding RNA-seq data (Man-13 R^2^ = 0.44 & Rebl.PAT R^2^ = 0.54).
