# Supplementary figures and images for: A secreted proteomic footprint for stem cell pluripotency
Source: PLoS One. 2024 Jun 14;19(6):e0299365. doi: 10.1371/journal.pone.0299365 (PMC11178176; doi:10.1371/journal.pone.0299365)

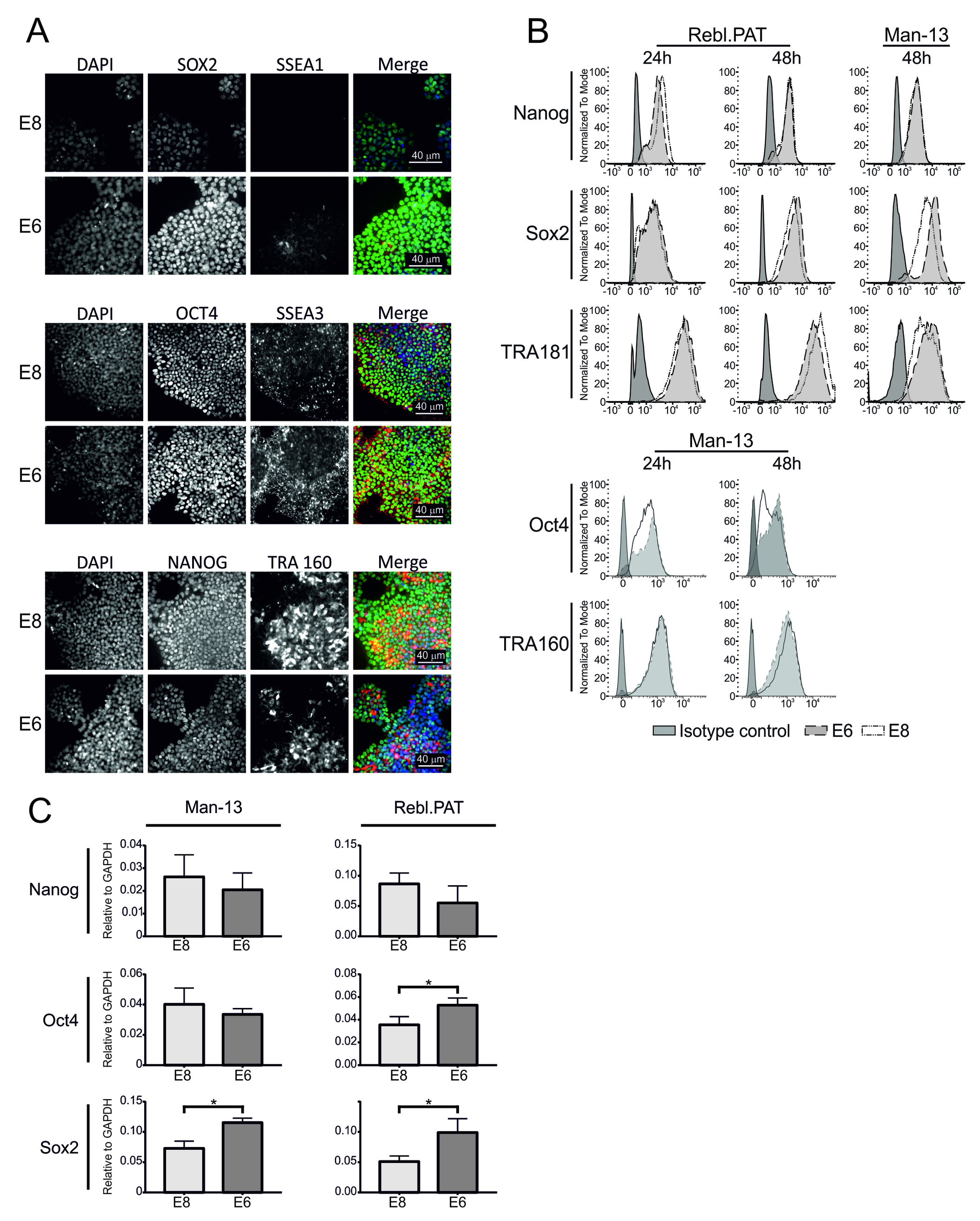

Supplement: S1 Fig — A) Immunocytochemistry of Man-13 cells cultured in E8 (control) or after 48 hours of culture in E6. B) Flow cytometry of Rebl.PAT and Man-13 cells cultured in E8, or after 48 hours of culture in E6. For Rebl.PAT cells, extra flasks were cultured alongside experimental flasks, and these were used for observation of cell state at the 24-hour time-point (shown for Rebl.PAT). C) QRTPCR of Man-13 and Rebl.PAT cells cultured in parallel to those used for medium collection, either maintained in E8, or after being incubated for 48 hours in E6. QRT-PCR for pluripotency associated markers Oct4 Sox2 and Nanog relative to GAPDH. (TIF) [file pone.0299365.s001.tif]

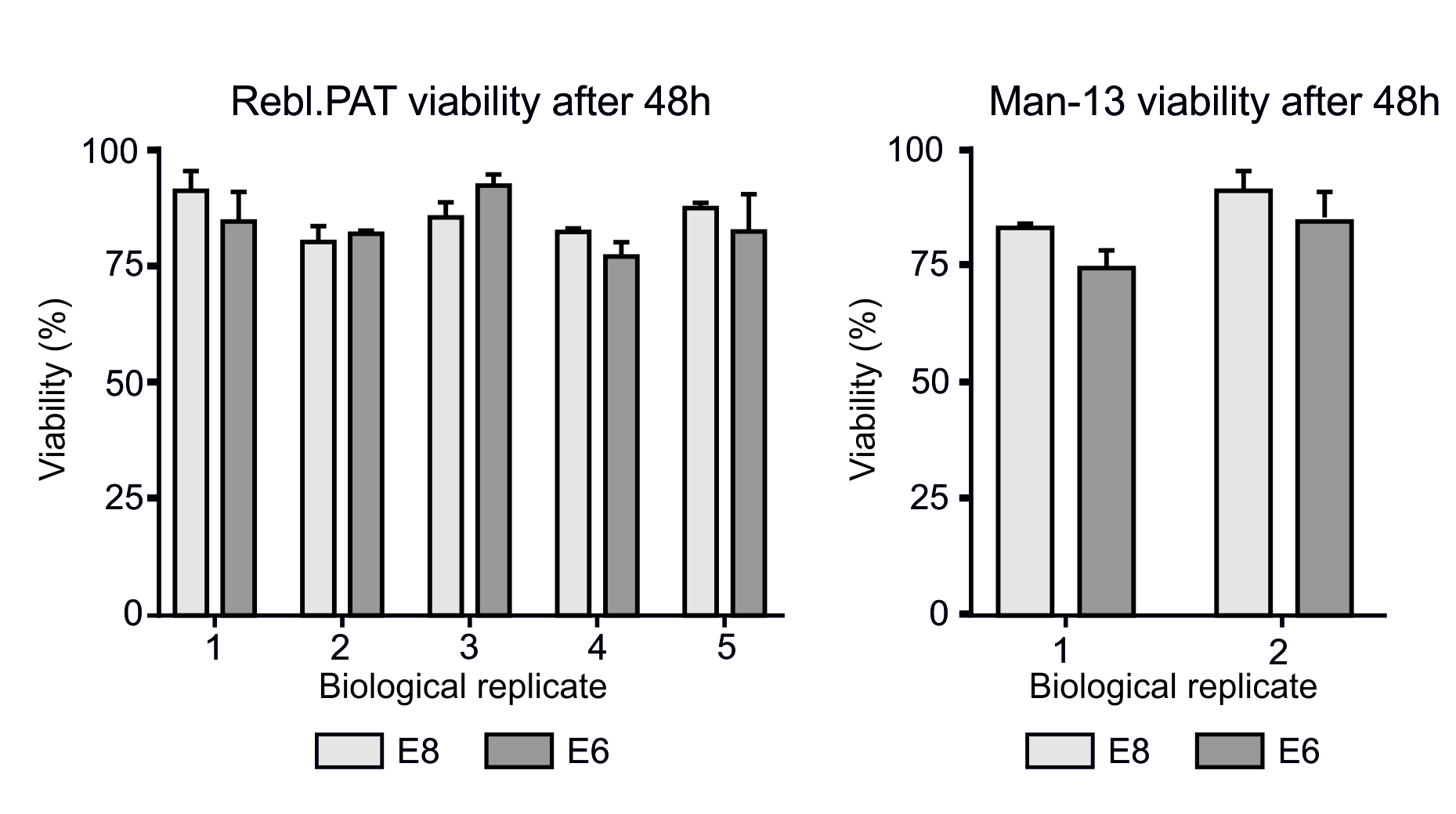

Supplement: S2 Fig — At the 48-hour timepoint (Fig 1), viability of E8 and E6 cultured cells was assessed using Via-1 cassettes (Chemometec). All three flasks of cells for each condition were assayed, and no significant change between the conditions was observed in any experiment. (TIF) [file pone.0299365.s002.tif]

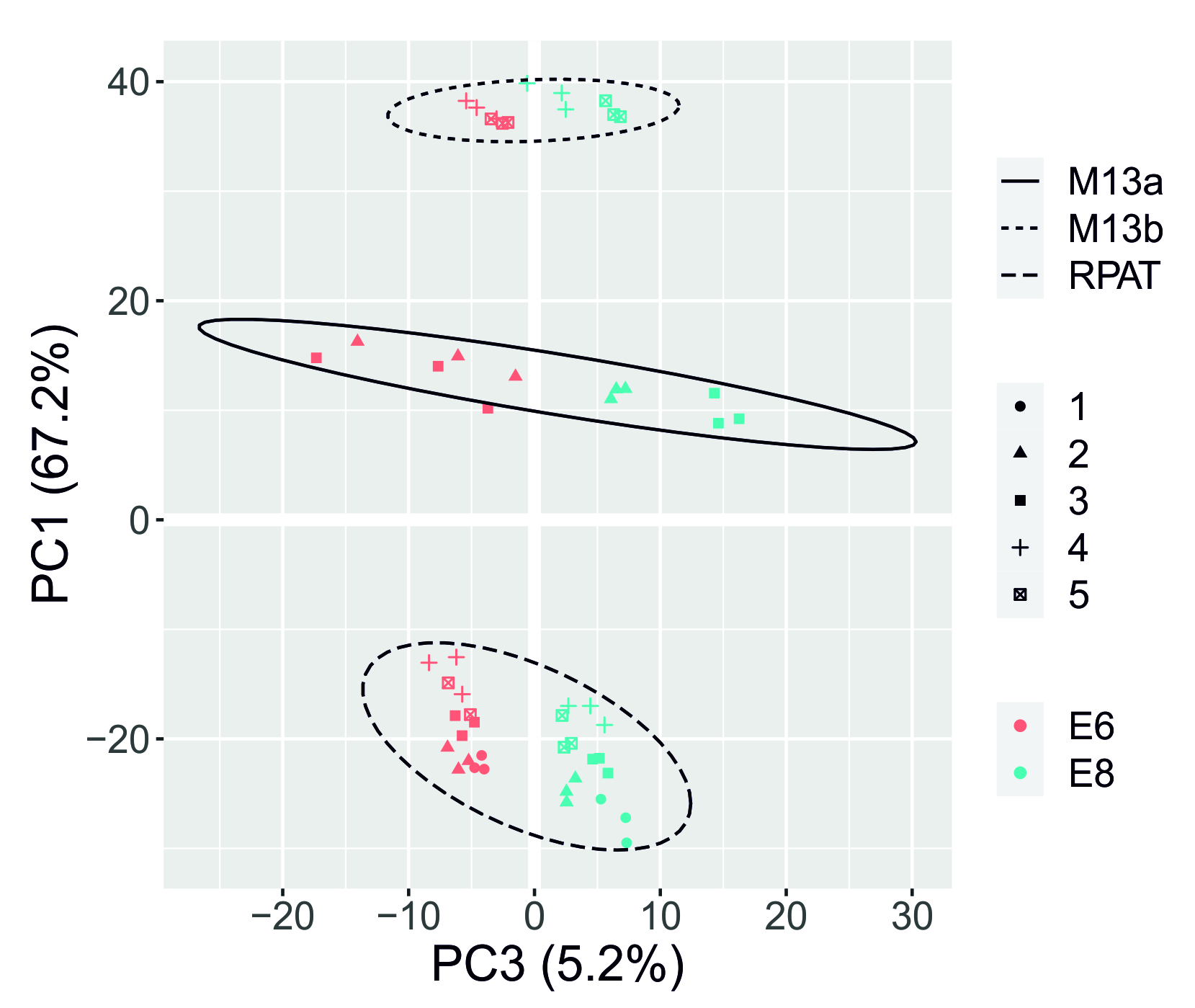

Supplement: S3 Fig — PC1 and PC3 are shown to demonstrate firstly the necessity of controlling for MS run in the statistical analysis (PC1, 67.2% of variance), and secondly that despite the other sources of variance, the experimental conditions are still clearly separated at PC3. (TIF) [file pone.0299365.s003.tif]

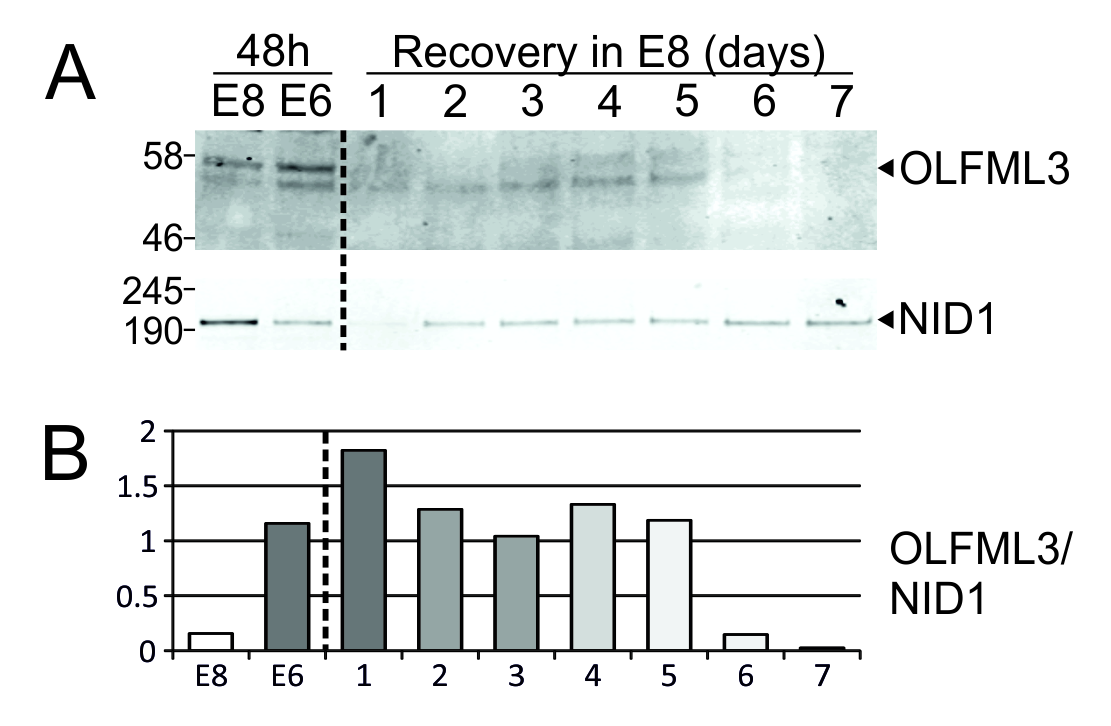

Supplement: S4 Fig — Man-1 cells were cultured under the same conditions as those in Fig 7, however the Western blot membrane was probed with OLFML3 & NID1 antibodies. Quantification shows the Log2 ratio of E6/E8 marker abundance (OLDML3/NID1). Densitometry was performed in ImageJ. Membranes were imaged using the LICOR Odyssey system. (TIF) [file pone.0299365.s004.tif]

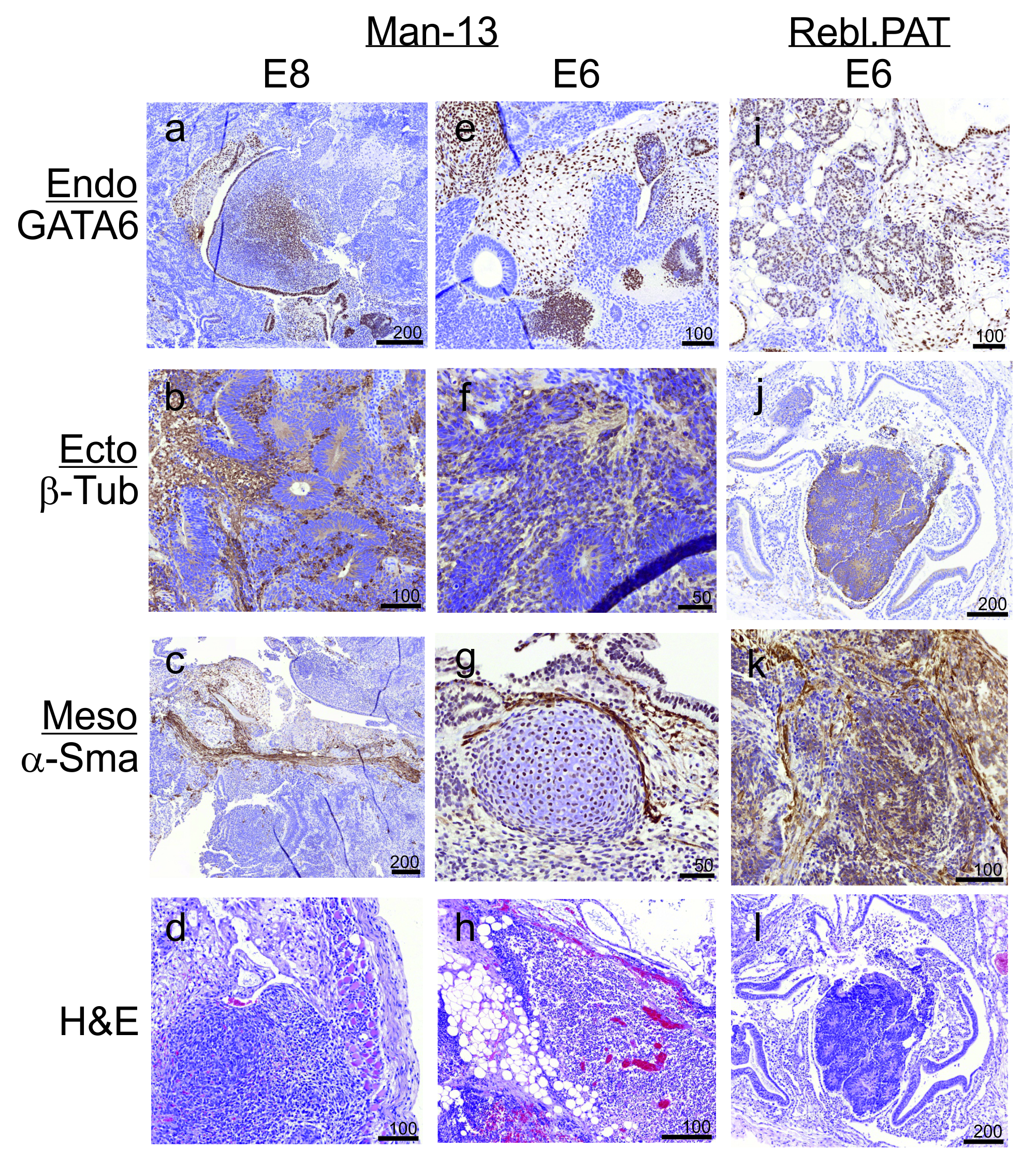

Supplement: S5 Fig — Paraffin sections were stained with Haematoxalin and eosin or with antibodies to early endoderm (GATA6) neurectoderm (Beta3 tubulin) or smooth muscle actin in mesoderm (alpha SMA) followed by a peroxidase labelled secondary antibody. Scale bar 100um. (TIF) [file pone.0299365.s005.tif]

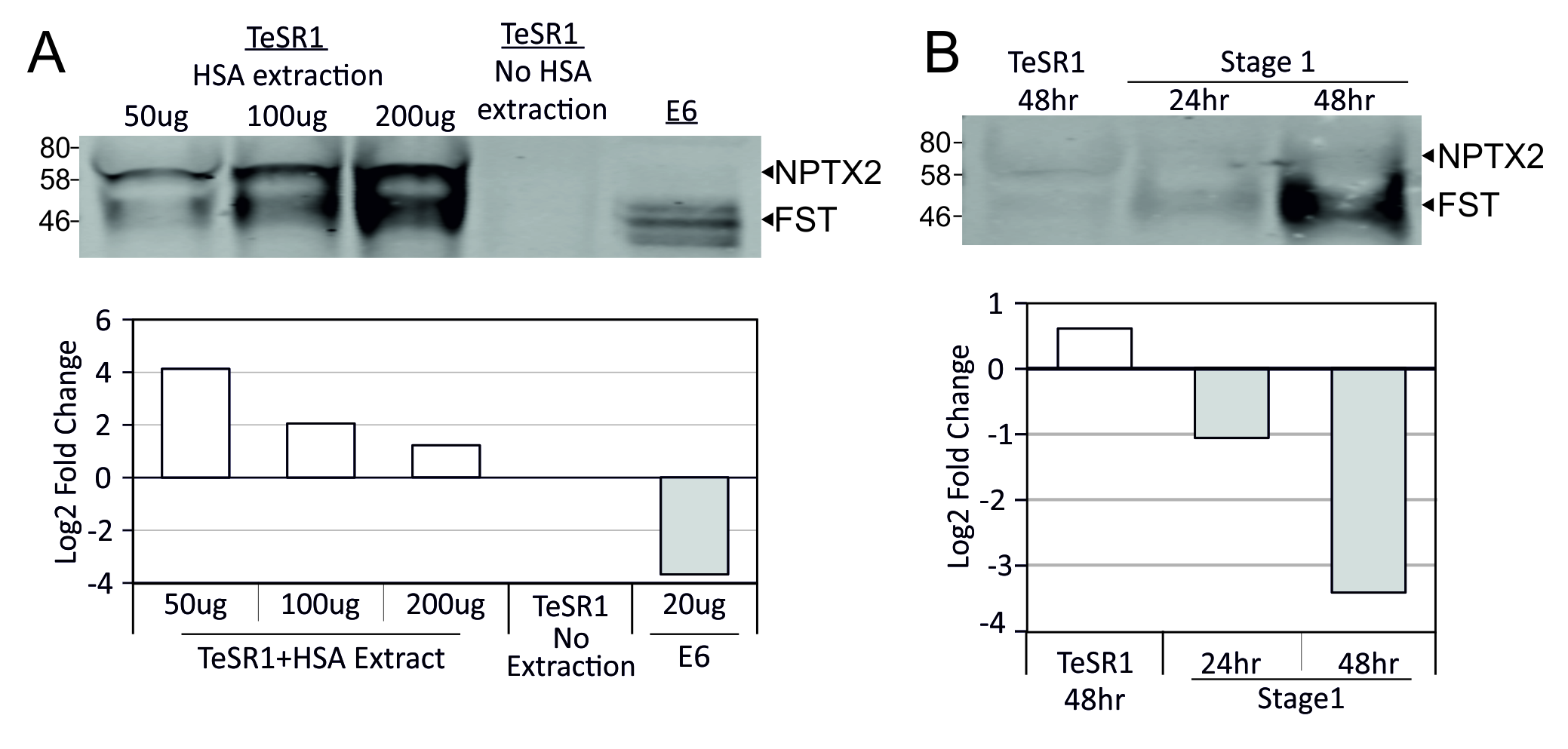

Supplement: S6 Fig — A) NPTX2 / FST Ratio of concentrated TeSR1 pluripotency media from cells grown for 48hrs, and medium subjected to HSA removal using 1%TCA-IPA or without HSA removal. B) NPTX2 / FST Ratio in media concentrated from cells grown in TeSR1 pluripotency medium versus mesodermal (stage1 chondrogenesis) differentiation medium showing that the change in protein ratios for NTPTX2 and Follistatin holds in both experiments if HSA is removed. (TIF) [file pone.0299365.s006.tif]

Fig. 6 A)

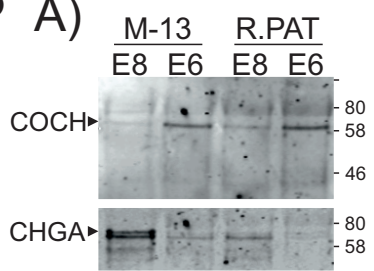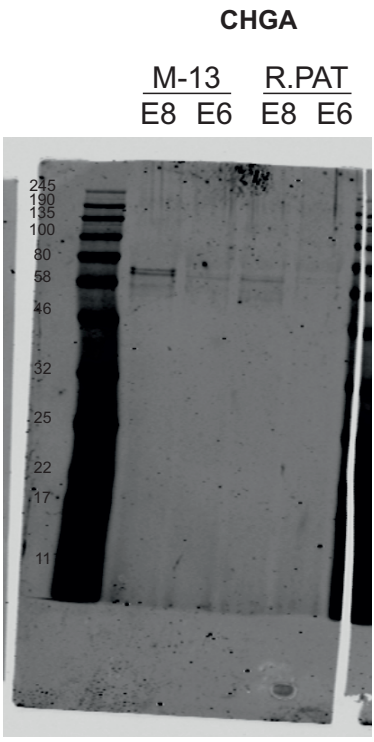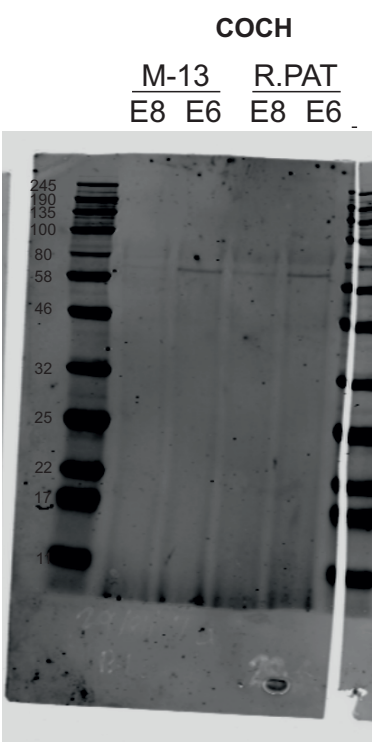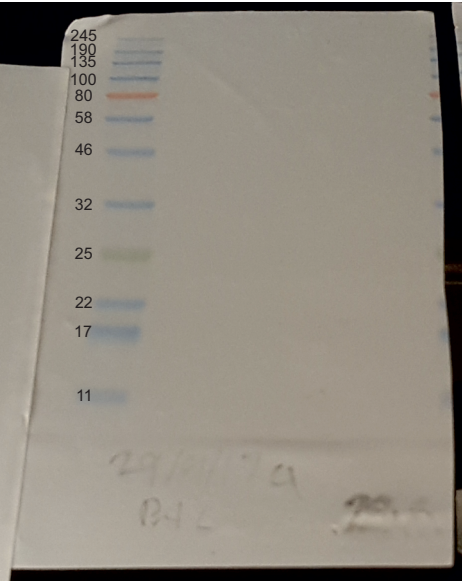

6.B)

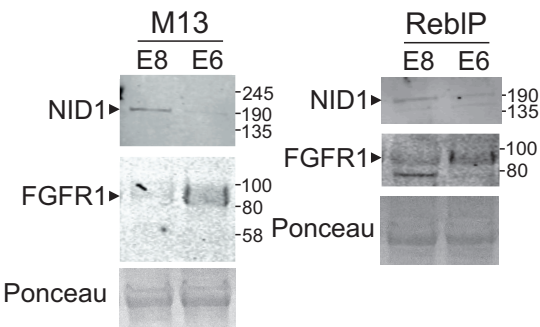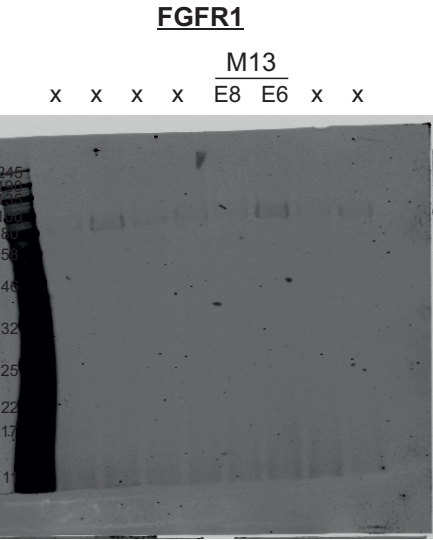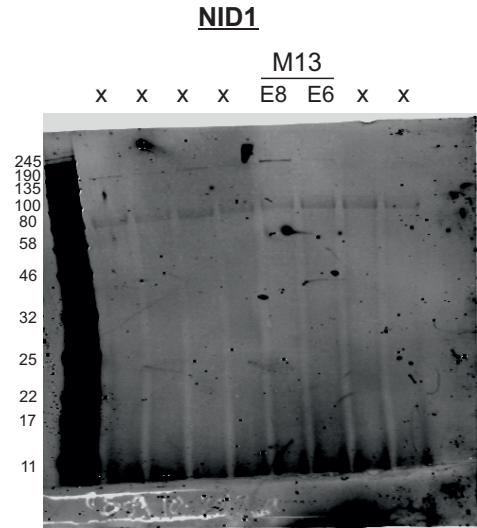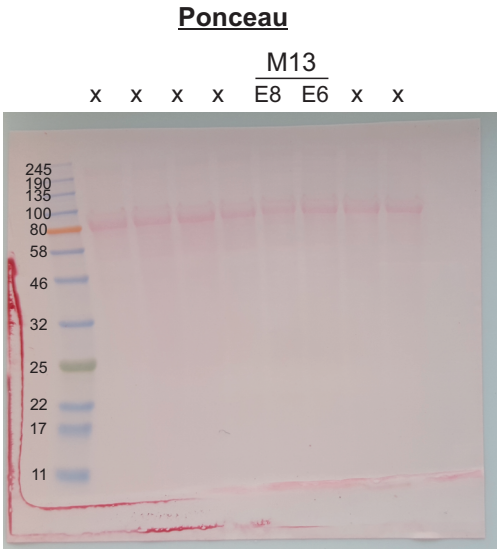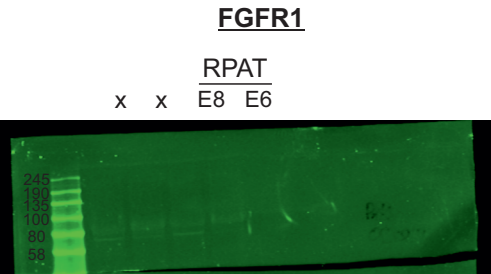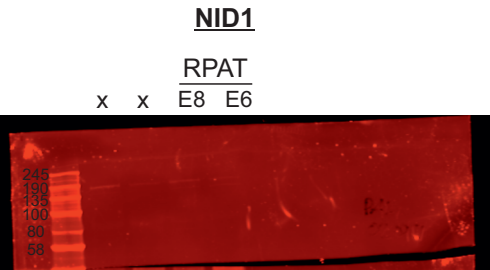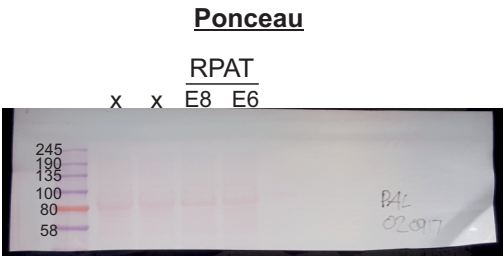

6.C)

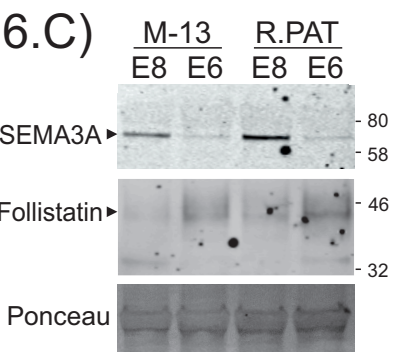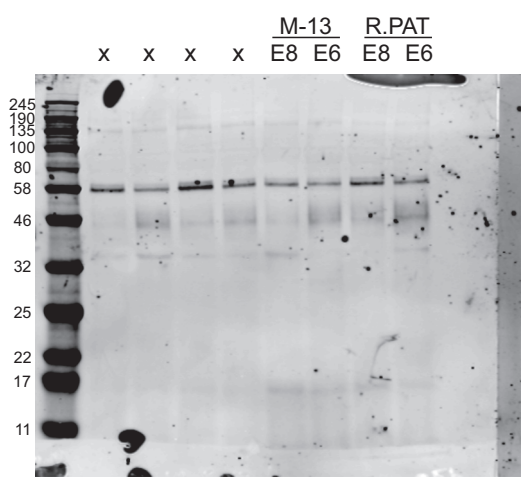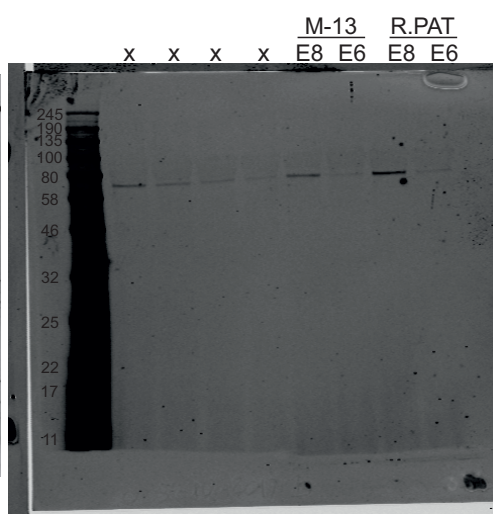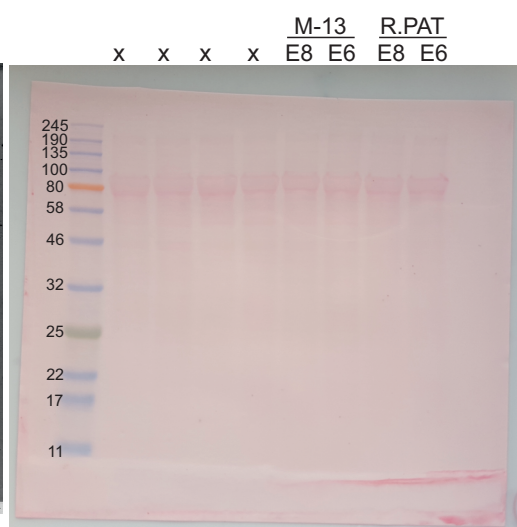

6.D)

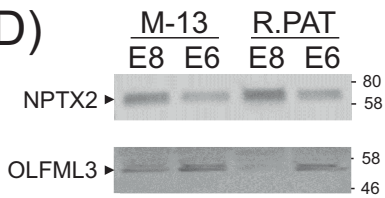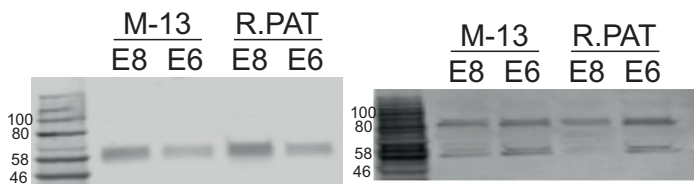

Fig. 7

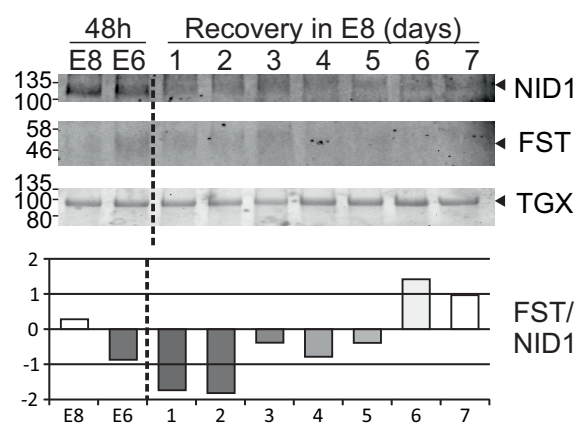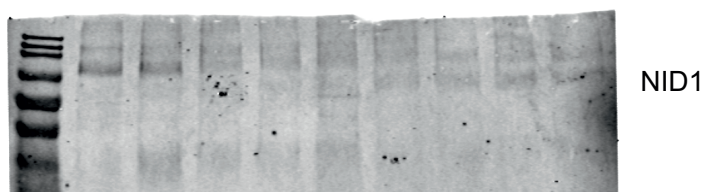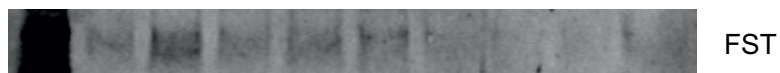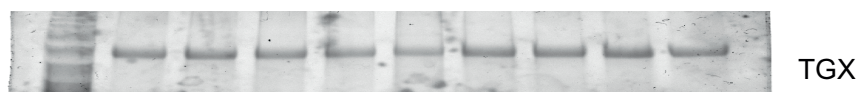

Fig. S4

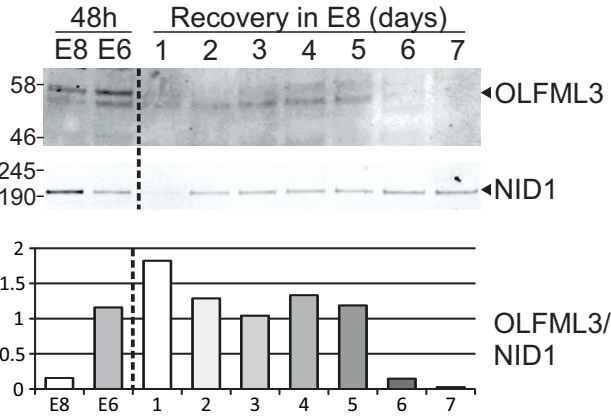

OLFML3

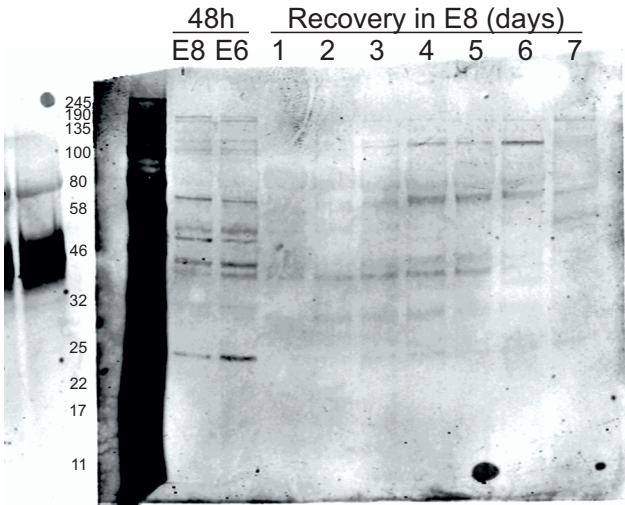

NID1

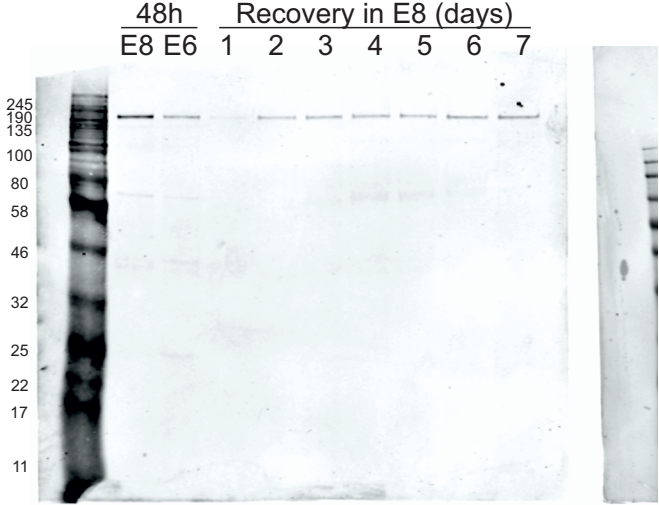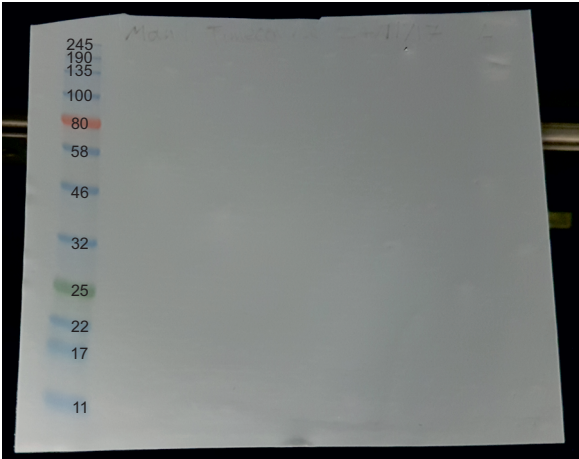

Supplement: S1 Raw images — (PDF) [file pone.0299365.s007.pdf]
